# Periodized versus non-periodized swimming training with equal total training load: Physiological, molecular and performance adaptations in Wistar rats

**DOI:** 10.1371/journal.pone.0239876

**Published:** 2020-09-30

**Authors:** Lucas D. M. Forte, Natália A. Rodrigues, André V. Cordeiro, Thais de Fante, Laís A. P. Simino, Adriana S. Torsoni, Márcio A. Torsoni, Claudio A. Gobatto, Fúlvia B. Manchado-Gobatto

**Affiliations:** 1 Laboratory of Applied Sport Physiology, School of Applied Sciences, University of Campinas, Limeira, São Paulo, Brazil; 2 Laboratory of Metabolic Disorders, School of Applied Sciences, University of Campinas, Limeira, Brazil; Medical University of Vienna, AUSTRIA

## Abstract

This study investigated the effect of non-periodized training performed at 80, 100 and 120% of the anaerobic threshold intensity (AnT) and a linear periodized training model adapted for swimming rats on the gene expression of monocarboxylate transporters 1 and 4 (MCT1 and 4, in soleus and gastrocnemius muscles), protein contents, blood biomarkers, tissue glycogen, body mass, and aerobic and anaerobic capacities. Sixty Wistar rats were randomly divided into 6 groups (n = 10 per group): a baseline (BL; euthanized before training period), a control group (GC; not exercised during the training period), three groups exercised at intensities equivalent to 80, 100 and 120% of the AnT (G80, G100 and G120, respectively) at the equal workload and a linear periodized training group (GPE). Each training program lasted 12 weeks subdivided into three periods: basic mesocycle (6 weeks), specific mesocycle (5 weeks) and taper (1 week). Although G80, G100 and G120 groups were submitted to monotony workload (i.e. non-modulation at intensity or volume throughout the training program), rodents were evaluated during the same experimental timepoints as GPE to be able comparisons. Our main results showed that all training programs were capable to minimize the aerobic capacity decrease promoted by age, which were compared to control group. Rats trained in periodization model had reduced levels of lipid blood biomarkers and increased hepatic glycogen stores compared to all other trained groups. At the molecular level, only expressions of MCT1 in the muscle were modified by different training regimens, with MCT1 mRNA increasing in rats trained at lower intensities (G80), and MCT1 protein content showed higher values in non-periodized groups compared to pre-training and GPE. Here, training at different intensities but at same total workload promoted similar adaptations in rats. Nevertheless, our results suggested that periodized training seems to be optimize the physiological responses of rats.

## Introduction

Chronic exercise results in positive adaptations that occur throughout the body. This involves molecular adaptations including changes in gene and protein expression levels, which are regulated by metabolic processes [1]. However, since it is difficult to reproduce human experiments due to variations in external (temperature, humidity, altitude) and internal factors (genetics, training status, nutrition, and others), animals have been used as models for the study of the physiological response to training [2, 3].

Despite the fact that use of an animal model provides a robust way to control independent variables, experiments with exercise commonly neglect to control for, and characterize, exercise volume and intensity. Thus, the “dose-response” to training intensity in this scenario remains unanswered. For example, some studies have compared the responses of animals to differing intensities of physical activities [4, 5], however, the failure of the studies to fully assess the volume (duration of exercise) of training performed by animals resulted in different total training loads being compared (a product of intensity and volume). This may have acted as a primary factor affecting data. An alternative way to study training intensity in an isolated way is to evaluate animals that have performed an equal total amount of work. This can be done by manipulating the volume of exercise performed by each group, as proposed in humans [6, 7].

The literature also lacks work in animals investigating the effect of modulating exercise intensity and volume (periodization) over a specific training period. In humans, periodization models are used to optimize gains associated with physical training [8]. In animals, few studies have employed a program involving the classically linearized periodization scheme initially proposed for humans [2, 9]. Araujo et al. [2] observed that physiological and performance adaptations occurred after swimming training, but no comparisons were made between the periodized and traditional (non-periodized) training models. Therefore, the effectiveness of periodization in the animal model remains unclear.

Training adaptations are preceded by the molecular modulation of mRNA and protein levels, which directly affect physiological machinery during exercise. For instance, monocarboxylate transporters (MCTs) have an intrinsic relationship with physical performance and adaptation to physical training. Among the 14 isoforms previously sequenced [10], MCT1 and 4 levels are most tightly associated with exercise [11]. Both act as symporters of lactate and regulate cellular compartment pH levels. As a consequence, MCTs 1 and 4 are intimately related to aerobic and anaerobic metabolism regulation [12]. The role of exercise in the regulation of expression levels of MCT genes and proteins are not completely known. In particular, the molecular pathways that regulate MCT availability have not been elucidated, further, the relationship between exercise volume and intensity, and gene and protein expression are also unknown [11].

Thus, the present study investigated the effect of non-periodized training performed at different intensities (80, 100 and 120% of the anaerobic threshold intensity) and a linear periodized training model adapted for swimming rats by assessing morphological, physiological, molecular and performance adaptations. To investigate the effects of different training programs, we evaluated the protein contents, blood biomarkers, tissue glycogen stores, body mass, and aerobic and anaerobic capacities of these rodents. At the molecular level, we measured the gene expression and protein contains of monocarboxylate transporters 1 and 4 (MCT1 and 4) in different tissues (soleus and gastrocnemius muscles), due these proteins are known to be involved in the adaptation to both, aerobic and anaerobic metabolic changes. In our hypothesis, despite being trained at different intensities, rats will adapt similarly to training program if the same total workload is maintained. On the other hand, the periodized training will significantly enhance the effectiveness of adaptation as a result of their exposure to varied stimuli.

## Methods

### Animals

Sixty young male Wistar rats (46 days-old and 277.3 ± 3.4 g when the experiment began) obtained from the Central Bioterium of the university were used in this study. Rats were housed in controlled environmental conditions (22 ± 1°C and 50 ± 5% relative humidity: five rats per cage) on a ventilated shelf with an air filtration system (ES2, ALESCO, BR). Lights in the room followed a 12:12 h light-dark cycle (lights on 06:00 to 18:00 h), and noise was kept to levels lower than 80 decibels on the shelf. Throughout all of the experiments, rats received filtered water and commercial balanced chow (CR1, NUVILAB, BR) *ad libitum*. Since rats are nocturnal, all handling procedures and exercise sessions were conducted during the dark period (~1 h after the lights had been turned off), and due to their high degree of light sensitivity, a red light (< 15 lux; > 600 nm) was used to reduce their exposure while allowing researchers to visualize and manipulate the animals [13].

All experimental procedures were conducted according to the recommendations of the Guide for the Care and Use of Laboratory Animals of the National Institutes of Health and the experimental design and procedures were approved by the Animal Use Ethics Committee of the University of Campinas (CEUA / UNICAMP protocol number: 3156–1). Euthanasia was performed by thoracotomy, after anesthesia by sodium thiopental, and this whole procedure was done to minimize the animal's suffering.

### Experimental design

**Fig 1:**
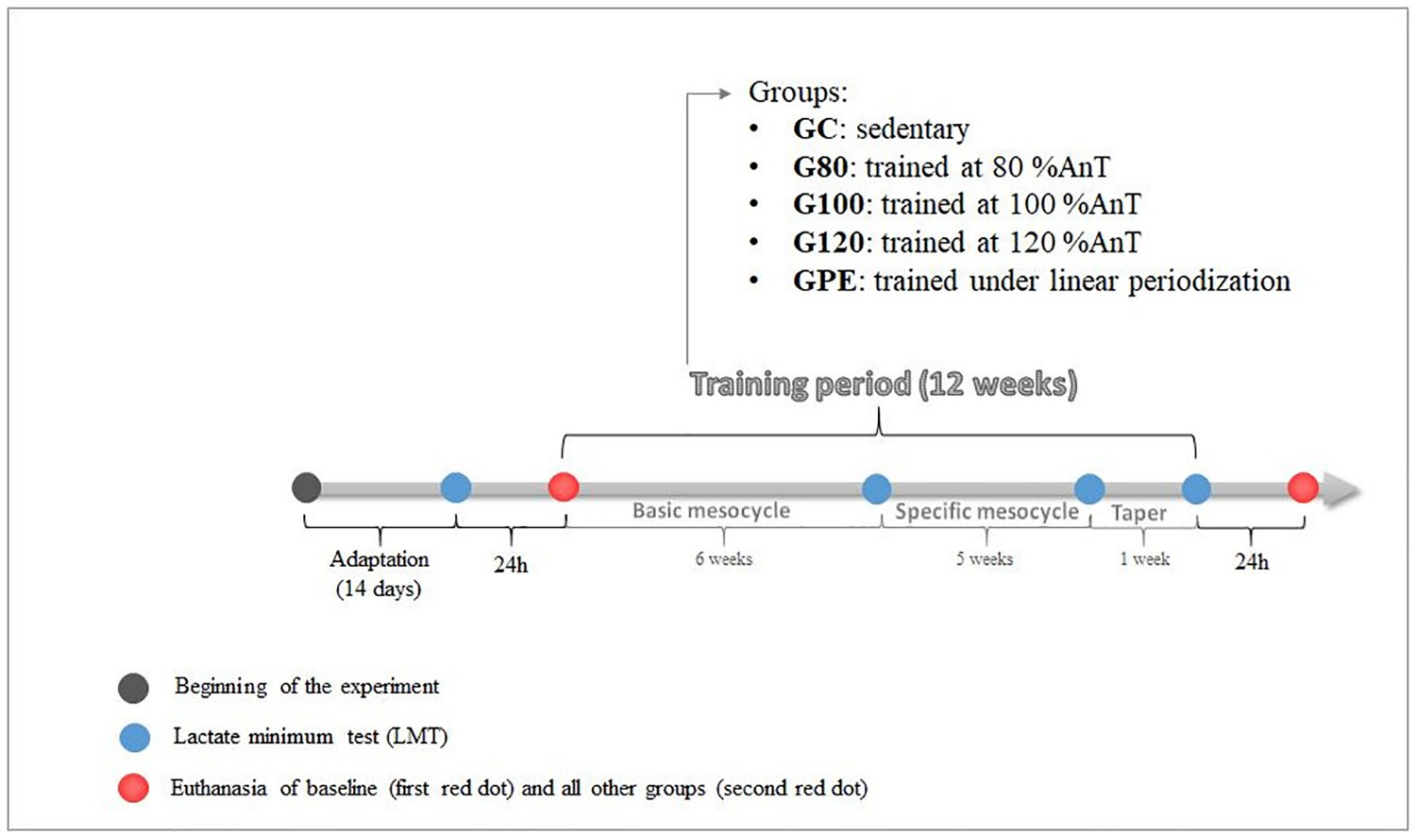
Schematic summary of the study design. After a 14-d adaptation period, rats were subjected to lactate minimum tests (LMTs) to determine the anaerobic threshold of animals, before the initiation of each training program and at the end of each mesocycle. Body mass, and food and water intake were measured weekly. Rats were euthanized 24 h after the last evaluation day.

After a 14-day adaptation to water and swimming exercise with loads, rats performed a lactate minimum test (LMT) for AnT determination. Thereafter, animals were randomly divided into six groups (n = 10 rats per group): a baseline group (BL; euthanized before training period), a control group (GC; not exercised during the training period), three groups exercised at intensities equivalent to 80, 100 and 120% of the AnT (G80, G100 and G120, respectively) and a group submitted to a linear periodized training program (GPE). Animals were subjected to the same procedures and environmental conditions, therefore, GC were submitted to a 30 s period of water exposure on during training days. Each training program lasted 12 weeks and was subdivided into three periods: basic mesocycle (six weeks), specific mesocycle (five weeks) and taper (one week). Although G80, G100 and G120 groups were submitted to monotony workload training (no modulation of intensity or volume throughout the training program), animals were evaluated during the same experimental timepoints as animals subjected to periodized training models to best be able to compare results of all programs. Additionally, at the end of each mesocycle, the LMT was performed. Body mass, food consumption and water intake were measured weekly. Rats were euthanized after being rested for 24 h after their last exercise session (after the taper period) and blood and tissue samples were collected for further analysis. Fig 1 contains a description of the study design.

### Adaptation to the aquatic environment

For all procedures, load was expressed as the percentage body mass (% bm) of the animal [14]. Loads were composed of lead and latex elastic and were tied to the back of each animal after a 14 d adaptation period, which was used to reduce stress and familiarize rats with the aquatic environment and swimming exercise regimens according to Lima et al. [15]. The adaptation process was composed of the following steps: i) three d in which animals were exposed to shallow water for 15 min (5 cm deep); ii) five days of swimming within deep tanks (100 cm deep) for an increasing duration that spanned from 2 to 10 min throughout a 6 d period; iii) Six days of applying a progressively increasing load (3 to 15% bm), while the duration of exercise decreased (5 min to 30 s).

Swimming throughout adaptation and training protocols was carried out in polyvinyl chloride tanks (30 cm diameter x 100 cm depth; one rat per tank) with water that was heated to 31 ± 1°C. The use of deep tanks with smooth surfaces prevented rats from being able to rest on the bottom of the tank and forced them to swim continuously [16].

### Training protocols

As previously described, G80, G100 and G100 groups performed exercise six d per week and their training program was made up of activities of fixed intensities and volumes. The daily volume of exercise (effort × duration) of G80, G100 and G120 were proportionally adjusted (37.5 min for G80, 30 min for G100 and 25 min for G120). As a result, the product of exercise volume (min) and intensity (%AnT) was the same for each group (for example, 120 x 25 = 3,000 a.u.; 80 x 37.5 = 3,000 a.u.). Animals of the GPE group were subjected to a linear periodized training program composed of three mesocycles (basic, specific and taper), which possessed four different exercise sessions, as follows:

1. **Endurance 1 (END1):** 60 min at 60% AnT
2. **Endurance 2 (END2):** 30 min at 100% AnT
3. **Endurance 3 (END3):** 3 sets of 5 min at 120% AnT separated by 1 min of passive recovery
4. **Anaerobic (ANA):** 5 exhaustive efforts at 260% AnT separated by 1 min of passive recovery

Variations in the exercise program were distributed along the mesocycles as previously described [2] and the training schedules of all groups are summarized in Table 1.

**Table 1:** Workload distribution throughout linear periodized training.

	Mon	Tue	Wed	Thu	Fri	Sat
**Basic Mesocycle**	**LMT**	END1	END2	END1	END2	END1
	END2	END1	END2	END1	END2	END1
	END1	END2	END1	END2	END1	END2
	END2	END3	END1	END1	END2	END3
	END2	END3	END1	END1	END3	END2
	END2	END3	END1	*Rest*	**LMT**	END2
**Specific Mesocycle**	END2	ANA	END1	END2	END1	END2
	END2	ANA	END1	END3	END1	ANA
	END2	ANA	END1	ANA	END1	ANA
	END2	ANA	END1	END3	END1	ANA
	END2	ANA	END1	*Rest*	**LMT**	END2
**Taper Mesocycle**	END2	ANA	END1	END3	*Rest*	**LMT**

LMT–Lactate minimum test; Adapted from de Araújo et al. [2]

### Lactate minimum test

The anaerobic threshold for each animal was determined using the LMT after a 24h rest period (for all groups). The protocol was described for humans and later adapted for swimming rats [17]. In summary, LMT measurements require an assessment of three phases: i) the induction to hyperlactemia after performing two, 30 s efforts while carrying 13% body mass (%bm) loads that are separated a 30 s rest period (the first effort lasted 30 s and the second effort was allowed to continue until the animal became exhausted); ii) a 9-min passive recovery period to allow for the accumulation of lactate in bloodstream and iii) an incremental phase in which 4.0, 4.5, 5.0, 5.5, 6.0, and 7.0% bm loads were carried for 5 min that were separated by 30 s intervals in which blood samples were collected and lactatemia was determined. The AnT was determined as intensity relative to a point derived from a second order polynomial curve. The time to exhaustion while carrying a 13% bm (Tex-13%) load was adopted as indicator of individual anaerobic capacity.

### Blood and tissue collection

Twenty-four hours after the last LMT, animals were euthanized while they were at rest. While euthanized, animals were not allowed to ingest food or water. Rats were anesthetized with sodium thiopental (25 mg × ml^-1^). A corneal and toe pinch was used to confirm adequate levels of anesthesia. After thoracotomy, blood samples were collected by cardiac puncture. The samples were centrifuged for 20 min at 2 000 rpm and the serum was stored at -80°C. Tissue samples from the liver, soleus, gluteus, and white and red portions of the gastrocnemius were dissected and immediately stored at -80°C for further analysis. Epididymal, retroperitoneal and brown adipose tissues were collected, weighed and discarded.

### Enzymes and metabolic analyses

Serum samples were used to determine levels of glucose, triglycerides, total cholesterol, high-density lipoprotein (HDL), low-density lipoprotein (LDL), total protein, albumin, uric acid, urea, creatinine, lactate dehydrogenase (LDH) and creatine kinase (CK). All analyses were performed using colorimetric methods using a spectrophotometer and specific kits (LABORLAB, BR). Free fatty acid (FFA) levels were determined using spectrophotometry according to Regouw et al. [18].

### Blood lactate analysis

To determine lactate concentrations within blood samples, 25 μL of blood was collected from the tip of the tail of each animal. Samples were deposited in a plastic tubes (1.5 ml) containing 400 μL trichloroacetic acid (4%) and were stored at 2 to 8°C. After centrifugation at 3 000 rpm for 3 min, supernatants were homogenized with a hydrazine hydrate-based reagent (88%; pH 9.45), EDTA, glycine, β-nicotinamide adenine dinucleotide (NAD) and LDH. Spectrophotometric measurements were performed using microplate reader (ASYS EXPERT PLUS UV, BIOCHROM, UK) at 340 nm and comparing sample values to a calibration curve [19].

### Determination of glycogen stores

Glycogen concentrations within skeletal muscle (red gastrocnemius and gluteus) and the liver were assessed as described in Dubois [20]. Muscle (250 mg) and liver (500 mg) samples were digested in KOH (30%), and mixed with saturated sodium sulfate and ethanol to precipitate glycogen. Samples were homogenized with 20 μL phenol (80%) and 2.0 ml sulfuric acid. Absorbance (490 nm) measurements were obtained after samples were boiled 15 min and concentrations of glycogen were calculated using a calibration curve.

### Real-time PCR analysis

Total RNA was extracted from tissues using Trizol reagent (INVITROGEN CORPORATION, CA, USA) in accordance with the manufacturer’s recommendations. Genomic DNA was removed from RNA samples via digestion with RNAse-free DNAse (RQ1, PROMEGA, MADISON, WI, USA). Reverse transcription was performed using total RNA from skeletal muscle (soleus and white gastrocnemius). Real-time PCR analysis of gene expression was performed using an ABI Prism 7500 Fast sequence detection system (APPLIED BIOSYSTEMS). MCT1 (Rn00562332_m1) and MCT4 (Rn00578115_m1) primers were obtained from Applied Biosystems. Optimal cDNA and primer concentrations, as well as the maximum efficiency of amplification, were obtained by performing a 5-point, 2-fold dilution curve analysis for each gene. Each PCR contained 20 ng of reverse-transcribed RNA and was run in accordance with the recommendations of the manufacturer of TaqMan PCR Master Mix (APPLIED BIOSYSTEMS). Levels of mRNA expression of target genes were normalized to levels of GAPDH and expressed as relative values using the comparative threshold cycle (Ct) method (2^-ΔΔCt^) according to the manufacturer’s instructions.

### Immunoblotting

Tissue samples were obtained and homogenized in freshly prepared, ice cold buffer [1% (v/v) Triton X-100, 0.1 mol/L Tris, pH 7.4; 0.1 mol/L sodium pyrophosphate; 0.1 mol/L sodium fluoride; 0.01 mol/L EDTA; 0.01 mol/L sodium vanadate; 0.002 mol/L PMSF; and 0.01 mg aprotinin/mL]. Insoluble materials were removed by centrifugation (10 000 × g) for 25 min at 4°C. Protein concentrations within supernatants were determined using the Biuret dye binding method. Supernatants were resuspended in Laemmli sample buffer and boiled for 5 min before separation via SDS-PAGE using a miniature slab gel apparatus (BIO RAD, RICHMOND, CA, USA). Electrotransfer of proteins from the gel to a nitrocellulose membrane was performed for 90 min at 120 V (constant). The membrane blots were probed with specific antibodies (anti-MCT1/bs-10249R and anti-MCT4/bs-2698R). Subsequently, blots were incubated with HRP-conjugated secondary antibodies (MERCK-MILLIPORE, AP307P). Results were visualized via chemiluminescence imaging (GENEGNOME XRQ, SYNGENE, USA) and band intensities were quantified using optical densitometry with ImageJ (1.50i) software. Band intensities were normalized to the loading control, α-tubulin.

### Measuring body mass, and food and water intake

The body mass of each rat was measured weekly on the night in which rats were not exercised. Food and water intake were measured when water bottles and chow containers were refilled, which occurred every two to three days, before the supply of food and water of each cage ran out. Because rats were housed collectively, data regarding food and water intake was calculated as an average value per kilogram (of all rats on each cage) per day.

### Data presentation and statistical analysis

Data are presented as mean ± standard error of mean (SEM). Normality and homogeneity were verified using Shapiro-Wilk and Levene tests, respectively. One-way ANOVA was used for the identification of different training programs on dependent variables obtained post-euthanasia (protein content, gene expression, blood biochemical parameters and tissue glycogen stores). Two-way ANOVA was applied to identify the effects of groups (intensities) and training periods on anaerobic threshold (AnT). When necessary, the Newman−Keuls post hoc test was used to detect the differences amongst groups. For all analysis, a 5% significance level was adopted.

## Results

### Aerobic capacity (anaerobic threshold)

**Fig 2:**
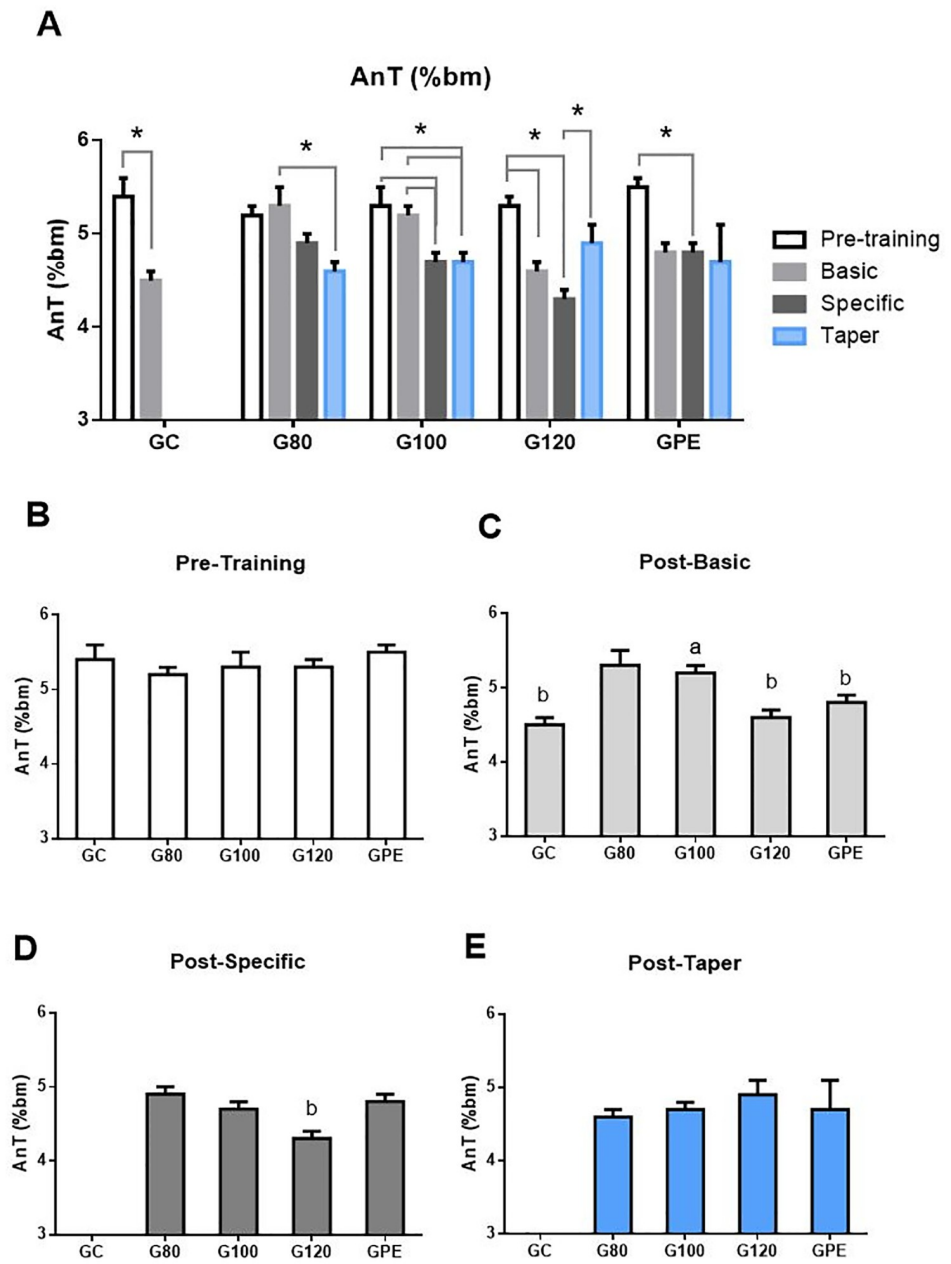
Anaerobic threshold intensity (AnT) compared between periods for each group (A), and between groups at pre-training (B) and after basic (C), specific (D) and taper (D) periods. Intensities are expressed as percentage of body mass (% bm), for all groups at pre-training and at the end of basic, specific and taper mesocycles. Linked bars indicate statistical significance *(P < 0.05); ^a^ P < 0.05 different to GC; ^b^ P < 0.05 different to G80.

Aerobic capacity (AnT intensity) data were expressed relative to body mass overload at pre-training timepoints and after each mesocycle (Fig 2). Regarding relative values, AnT values of the GC group significantly decreased after the basic mesocycle. After specific and taper, AnT could not be determined since animals of this group did not complete more than two incremental LMT stages (thus, their AnT intensity were lower than 4.0% of bm). For trained groups, relative AnT values of all groups significantly decreased at some point throughout the training process, however, this difference was not statistically significant by the end of taper mesocycle (except for G100).

### Total training workload

**Fig 3:**
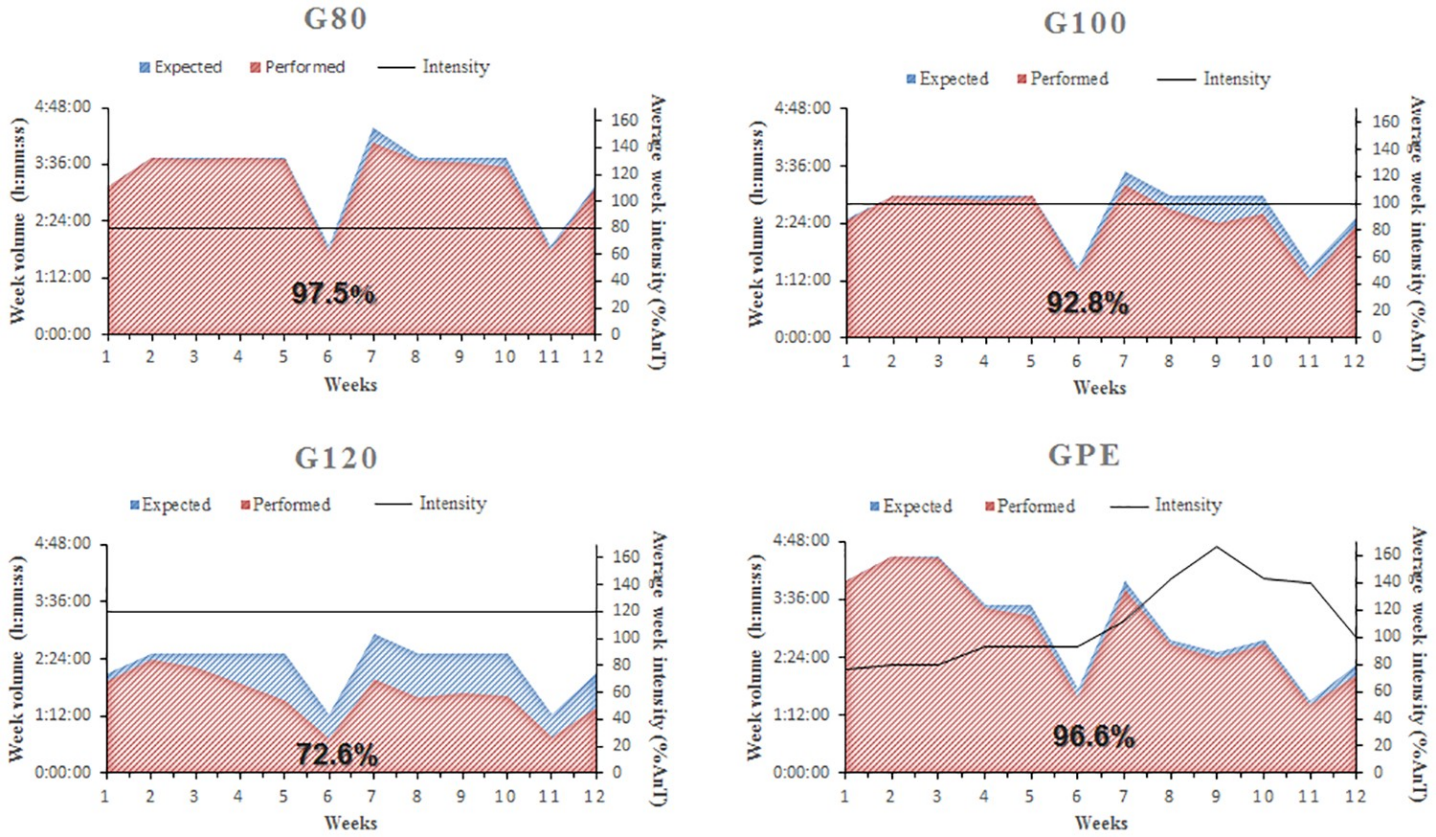
Ilustration of expected (blue) and performed (red) volumes and average exercise intensities (black line) during the 12-week training period for G80, G100, G120 and GPE groups.

When the exercise intensity of each day of training was fixed, the workload of each animal varied according to the total volume (duration) of exercise performed. Expected (blue) and performed (red) exercise volumes of each group during the 12-week training period are illustrated in Fig 3. With the exception of G120, all trained groups completed more than 90% of the expected training volume. G120 could perform only 72.6% of total expected volume, which resulted in a significantly (P < 0.05) reduced total workload of training (product of intensity and total volume).

### Body mass, food and water intake

**Fig 4:**
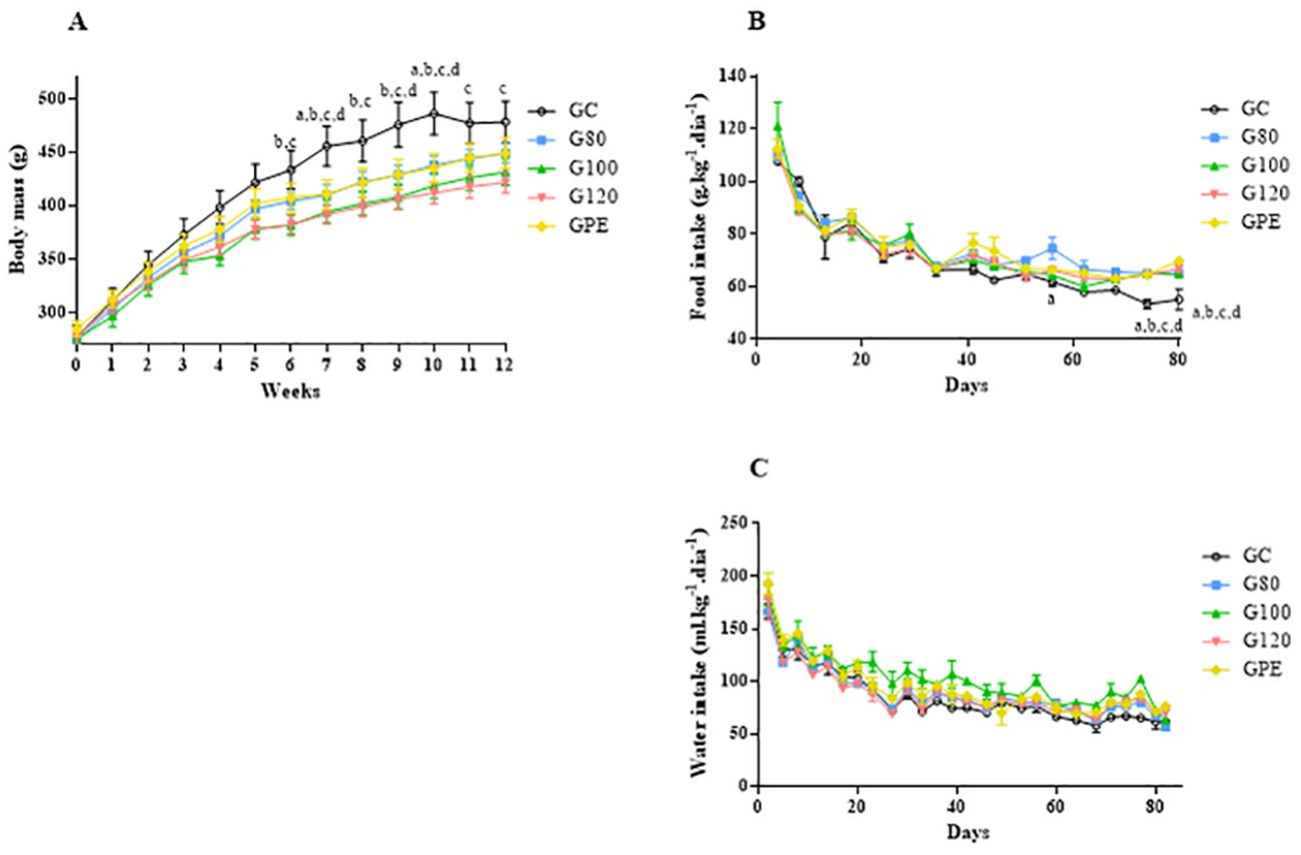
Graphic representation of body mass (A), food (B) and water (C) intake values during the training period for all groups. ^a^ P < 0.05 different to G80; ^b^ P < 0.05 different to G100; ^c^ P < 0.05 different to G120; ^d^ P < 0.05 different to GPE.

Mean ± SEM of weekly body mass measurements and daily food and water intake values are shown in Fig 4A–4C. The body mass of animals increased throughout the training period, however, only the GC group was significantly increased (P < 0.05) relative to the other groups. These differences were first observable at the 6^th^ week (end of basic mesocycle). Food and water intake of all groups were reduced during training period, and no differences were observed between trained and control groups (Fig 4B and 4C). The GC group had significantly lower food intake levels than the G80 on day 56, and from all trained groups on days 74 and 80 (P < 0.05).

### Epididymal, retroperitoneal and brown adipose tissue

**Fig 5:**
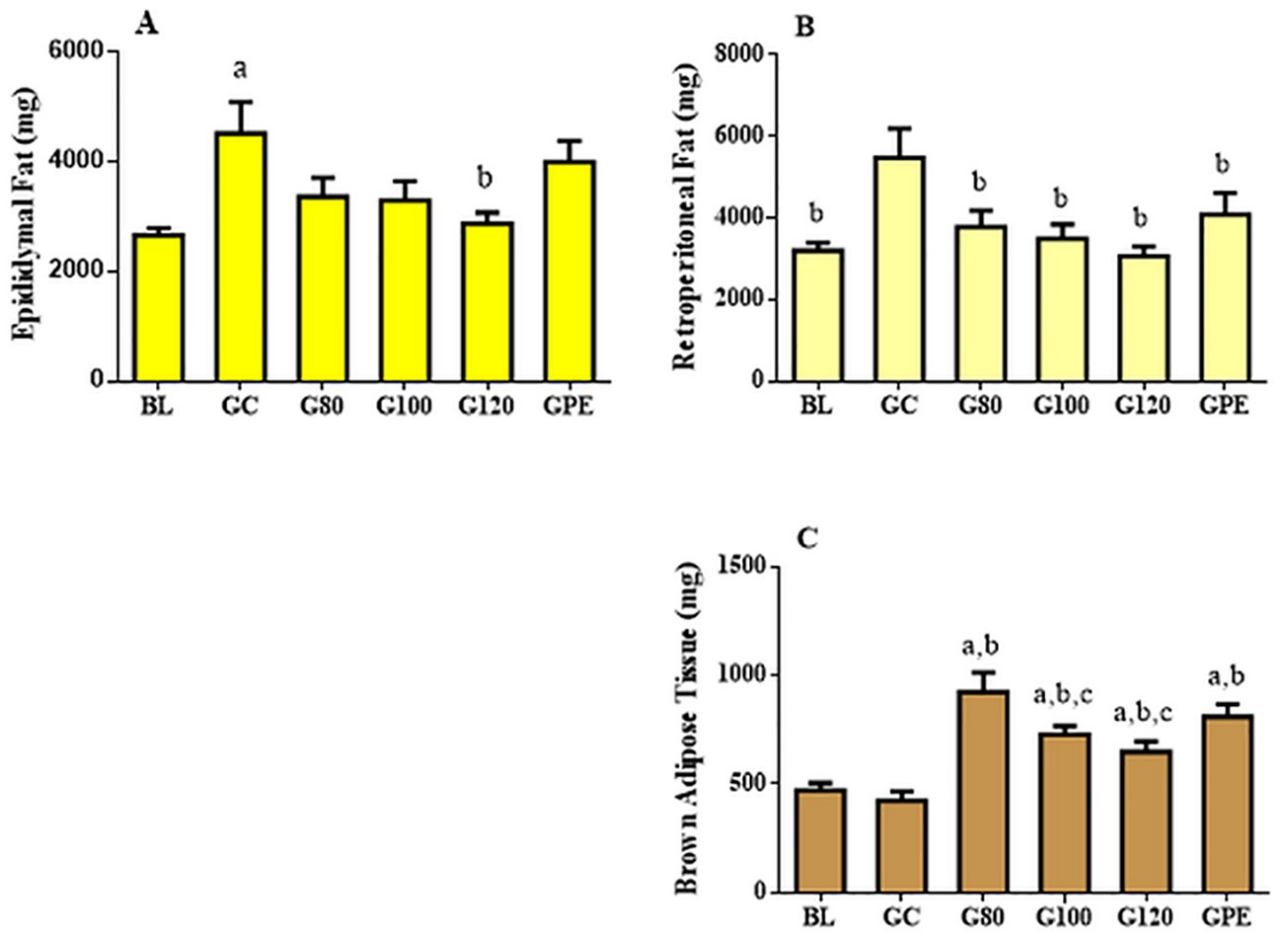
Mean ± SE weights (g) of epididymal (A), retroperitoneal (B) and brown adipose tissues (C) from each experimental group. ^a^P < 0.05 *vs* BL; ^b^P < 0.05 *vs* GC; ^c^P < 0.05 *vs* G80.

Epididymal adipose tissue was less affected by training than other types (Fig 5A). Increased levels were only observed when the GC group was compared to baseline and G120 values (P < 0.05). As for retroperitoneal tissue, all trained groups managed to maintain similar baseline levels. Values measured in trained groups were reduced (P < 0.05) when compared to the GC group (Fig 5B). In contrast, brown adipose levels for all trained groups were significantly increased relative to levels observed in GC and BL groups (Fig 5C). Brown adipose tissue (BAT) levels in G80 were statistically greater than those of G100 and G120 groups.

### Blood biomarkers

Concentrations of biomarkers within blood samples are presented in Table 2. All trained groups and the GC had reduced levels of glucose after training periods when compared to BL values (P < 0.05). Only G80 and G100 groups had total cholesterol levels that were significantly reduced compared to BL values. Albumin levels, in contrast, were increased in G120 and GPE *vs* G80 and G100 (P < 0.05). All trained groups presented statistically reduced uric acid and creatine kinase values when compared to the GC group. Despite the fact that LDL levels were decreased in all trained groups, only mean LDL values of the GPE group were significantly lower than BL values and those of the GC group. Levels of FFA was also reduced in GPE and G100 groups *vs* BL (P < 0.05).

**Table 2:** Mean ± SE of serum metabolic parameters after training period for all groups.

	BL	GC	G80	G100	G120	GPE
**GLU (mg/dL)**	108.8 ± 2.8	85.1 ± 5.5 ^a^	80.0 ± 2.9^a^	84.3 ± 4.9 ^a^	69.8 ± 3.4^a^	85.8 ± 5.5^a^
**CHOL (mg/dL)**	72.9 ± 3.6	71.8 ± 5.1	53.1 ± 6.1^a^	54.7 ± 3.6 ^a^	61.6 ± 2.6	57.9 ± 5.0
**TG (mg/dL)**	53.6 ± 7.9	46.1 ± 8.1	40.7 ± 5.4	57.1 ± 7.1	58.1 ± 5.4	57.2 ± 3.2
**TP (g/dL)**	3.7 ± 0.3	4.0 ± 0.2	3.7 ± 0.2	3.9 ± 0.2	3.7 ± 0.2	4.7 ± 0.6
**ALB (g/dL)**	3.4 ± 0.1	3.0 ± 0.1	3.3 ± 0.1	3.0 ± 0.2	3.7 ± 0.2^b^^,^ ^d^	3.7 ± 0.1^b^^,^ ^d^
**UA (mg/dL)**	0.8 ± 0.1	0.9 ± 0.1	0.5 ± 0.1^b^	0.4 ± 0.1^b^	0.4 ± 0.0^b^	0.4 ± 0.1^b^
**LDH (U/L)**	459.3 ± 49.2	643.7 ± 73.7	598.7 ± 51.6	617.6 ± 81.2	528.9 ± 44.1	570.7 ± 53.8
**CK-MM (U/L)**	238.7 ± 25.6	409.5 ± 63.1^a^	194.1 ± 16.0^b^	199.5 ± 14.9^b^	181.9 ± 21.4^b^	199.8 ± 23.^b^
**CREA (mg/dL)**	0.4 ± 0.0	0.3 ± 0.0	0.4 ± 0.0	0.4 ± 0.0	0.5 ± 0.1	0.5 ± 0.0
**URE (mg/dL)**	56.7 ± 2.8	49.9 ± 1.8	55.2 ± 1.5	49.4 ± 2.2	54.6 ± 3.7	51.7 ± 2.6
**HDL (mg/dL)**	29.7 ± 7.0	24.3 ± 6.8	32.5 ± 7.7	18.8 ± 0.9	41.6 ± 11.2	39.5 ± 9.7
**LDL (mg/dL)**	33.9 ± 5.5	44.6 ± 4.3	25.0 ± 6.0	23.7 ± 4.1	29.8 ± 2.4	14.3 ± 4.7^a^^,^ ^b^
**FFA (μEq/L)**	275.2 ± 47.4	245.7 ± 35.6	165.4 ± 28.5	142.3 ± 26.6^a^	137.8 ± 17.5	119.1 ± 34.3^a^

Abbreviations: Glucose (GLU), total cholesterol (CHO), triglycerides (TG), total protein (TP), albumin (ALB), uric acid (UA), lactate dehydrogenase (LDH), creatine kinase (CK), creatinine (CREA), urea (URE), high density lipoprotein (HDL), low density lipoprotein (LDL) and free fatty acid (FFA).

^a^ P < 0.05 relative to BL;

^b^ P < 0.05 relative to GC;

^d^ P < 0.05 relative to G100.

### Glycogen stores

**Fig 6:**
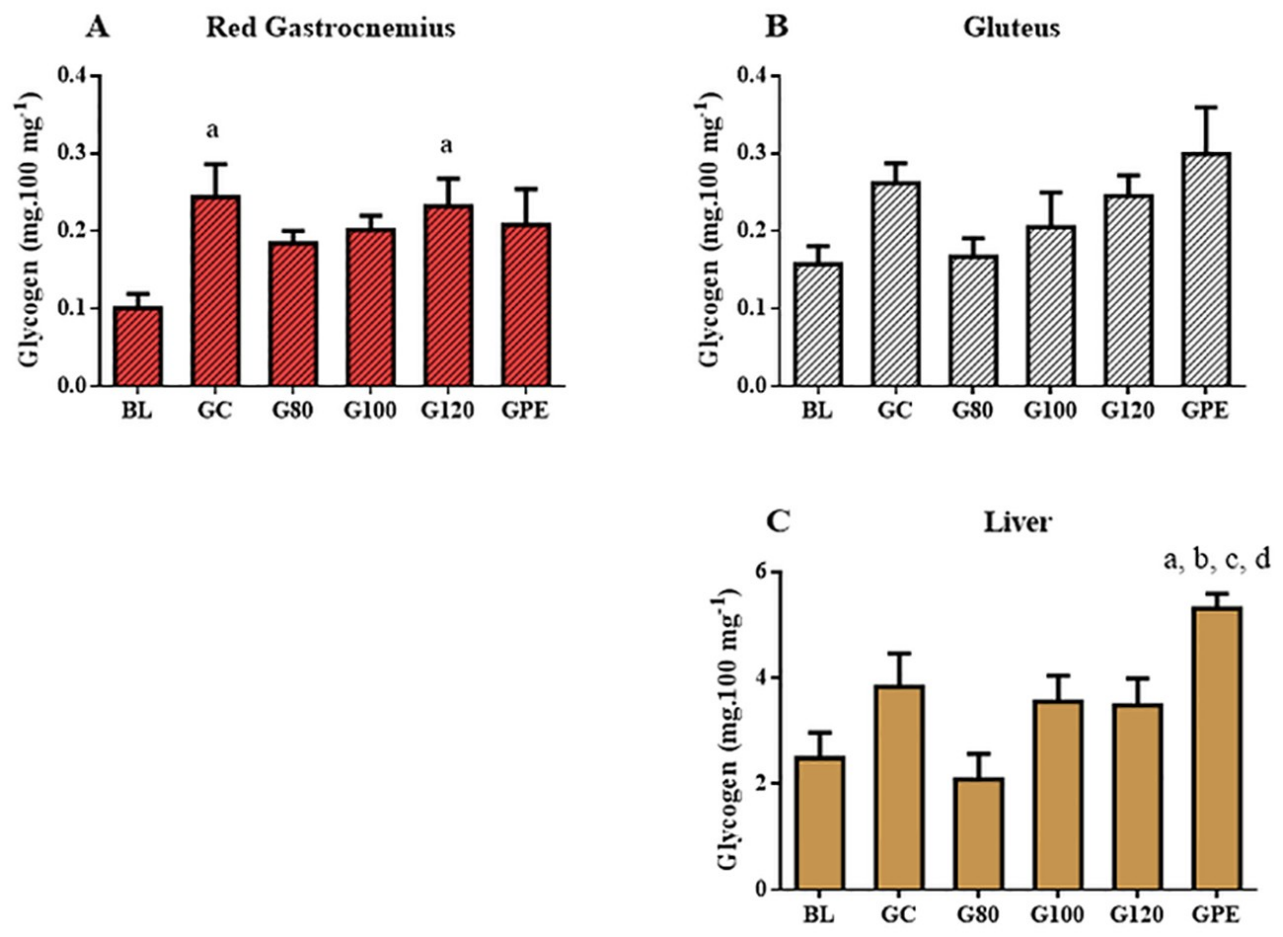
Glycogen content in red gastrocnemius (A), gluteus (B) and the liver (C) of all experimental groups. ^a^P < 0.05 *vs* BL; ^b^P < 0.05 *vs* GC; ^c^P < 0.05 *vs* G80; ^d^P < 0.05 *vs* G100.

Glycogen stores were not affected by training in predominantly oxidative (Fig 6A) and glycolytic muscles (Fig 6B). Glycogen stores within the red portion of the gastrocnemius were increased in GC and G120 groups when compared to BL values. However, these differences seemed to be age-related since the mean values of other trained groups were also higher than BL (despite the fact that they were not statistically significant). Interestingly, significant increases in hepatic glycogen stores were observed exclusively in rats trained using the linear periodized model when compared to BL, GC, G80 and G100 (Fig 6C).

### Levels of gene and protein expression

**Fig 7:**
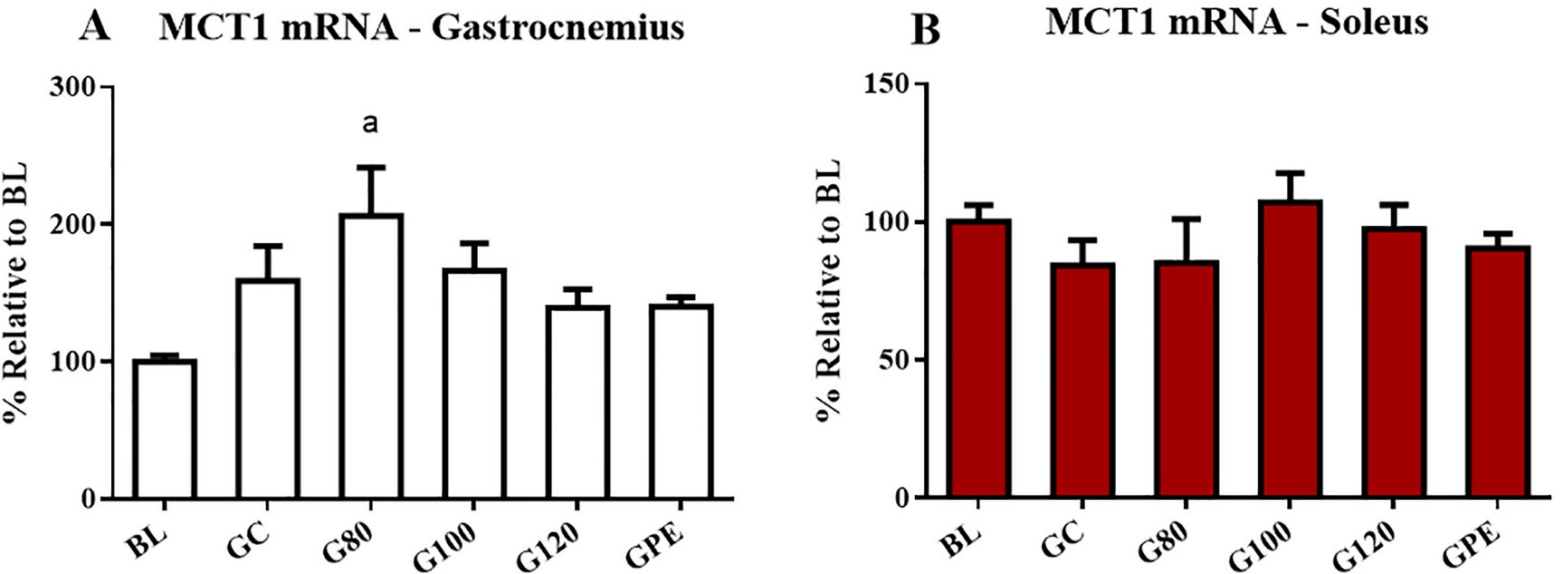
MCT1 mRNA expression levels in the gastrocnemius (A) and soleus (B) of all experimental groups. ^a^ P < 0.05 different to BL.

**Fig 8:**
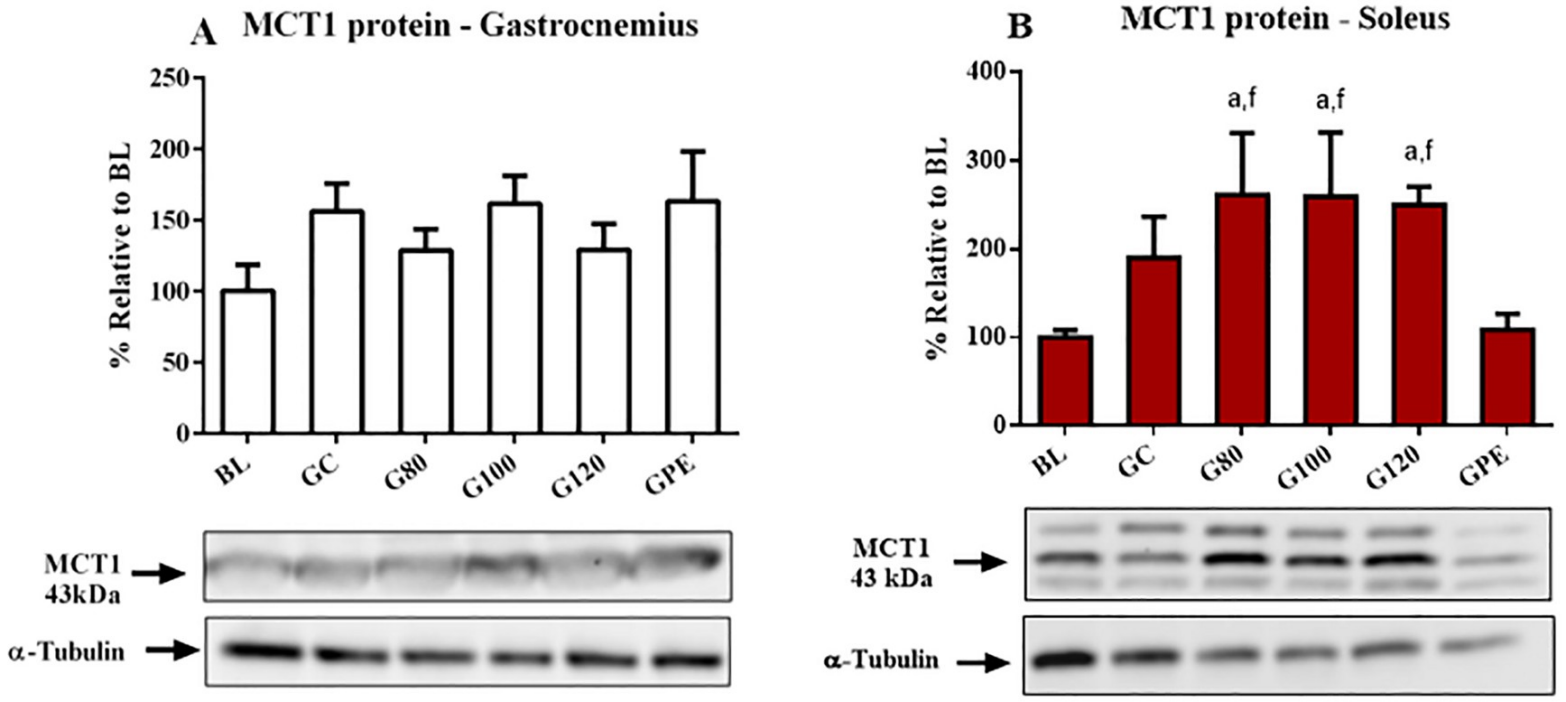
MCT1 protein expression levels in the gastrocnemius (A) and soleus (B) of all experimental groups. ^a^ P < 0.05 different to BL; ^f^ P < 0.05 different to GPE.

**Fig 9:**
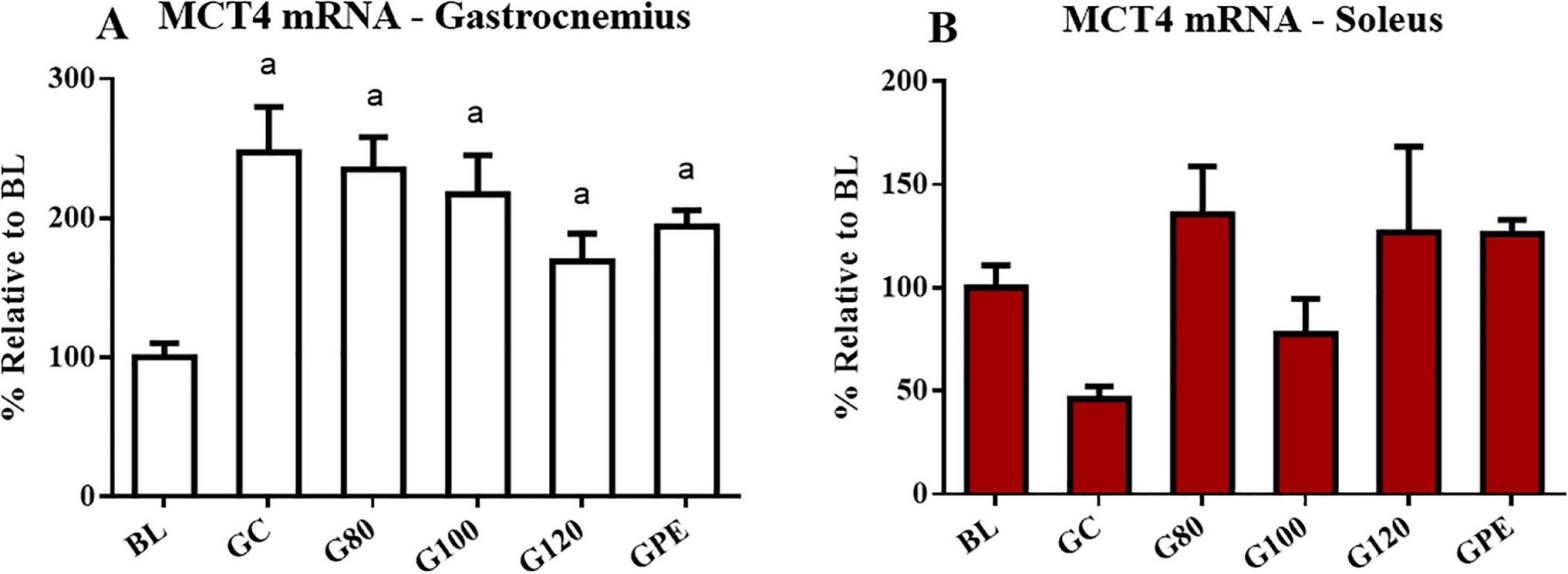
MCT4 mRNA expression levels in the gastrocnemius (A) and soleus (B) of all experimental groups. ^a^ P < 0.05 different to BL.

**Fig 10:**
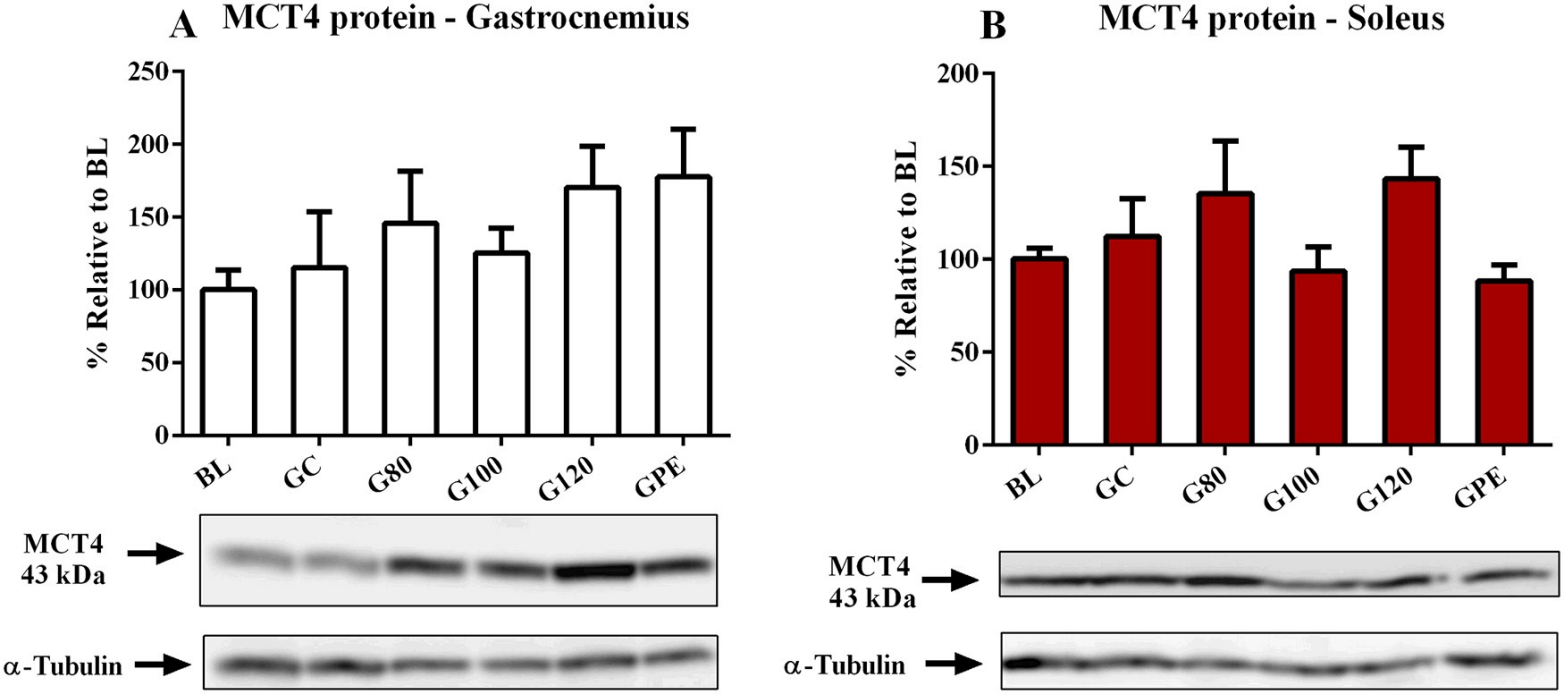
MCT4 protein expression levels in the gastrocnemius (A) and soleus (B) of all experimental groups.

Expression levels of MCT genes were not influenced by exercise. Some differences in levels were likely age-related, such as increased MCT4 mRNA levels observed in the gastrocnemius that occurred in trained and non-trained animals (Fig 7A). Only MCT1 mRNA in gastrocnemius seemed to respond to physical training. Levels observed for the G80 group were increased relative to BL (Fig 7A). Regarding protein content, only MCT1 in the soleus was significantly increased (P < 0.05) in groups not subjected to periodized training (G80, G100 and G120) in comparison to BL and GPE (Fig 8B). Figs 9 and 10, respectively, show the results of the gastrocnemius muscle for MCT4 protein content and mRNA expression.

## Discussion

Our results revealed that subjecting rats to the training models (periodized or non-periodized) resulted in significant adaptation to exercise at molecular, morphological and physiological levels. No differences were observed among animals that were subjected to non-periodized training regimes. The response of the GPE group to training was optimized. After training, the group had increased hepatic glycogen stores and reduced blood lipid levels, which confirms our initial hypothesis.

### Aerobic and anaerobic performance

The aerobic capacity (AnT) of animals of the GC group after the basic mesocycle were significantly decreased relative to the pre-training period. At the end of specific mesocycle and taper, LMT could not be used to determine AnT in animals of the GC group. It is possible that the AnT of the GC group decreased to below 4% bm (initial workload during incremental stage of LMT), which resulted in the continuous accumulation of blood lactate and the absence of the U-shaped curve required for the determination of AnT [17]. In two different studies, de Araujo [2, 4] observed declines in aerobic capacity after periodized and non-periodized swimming training at intensities near the AnT of rats, and subjecting animals to chronic exercise only attenuated these declines in comparison to control groups. The authors suggested that the limited space within conventional cages provides animals with a compulsory sedentary environment, which may inhibit the adaptation to training. Accordingly, it has been shown that providing animals with a wide housing space can enhance the physical fitness levels and adipose morphologies of trained rats [21]. AnT levels in all trained rats were significantly decreased after basic and/or specific mesocycles when compared to the pre-training period. However, at the end of the taper period, these reductions were not statistically significant for most groups. This indicates that exposure to chronic exercise prevented aerobic capacity reductions that have been commonly observed in other studies [2, 4, 22].

One of the main goals of the present study was to investigate the isolated effects of exposure to different training intensities in animals subjected to equal external workload levels. Animals of the G80, G100 and GPE groups completed 97.5, 92.8 and 96.6% of the total volume expected, respectively. However, rats of the G120 completed only 72.6% of the total volume expected. This resulted in a significantly reduced total workload of training when compared to other groups. This suggests that the internal workload increases proportionally to the external workload until the AnT is reached. While above AnT, the external workload has a significantly enhanced impact on internal workload. This hypothesis was supported by data showing that above AnT, blood lactate levels increase and intracellular pH decreases disproportionally to exercise intensity, which leads to hyperventilation and subsequent exercise interruption [23]. This finding which explains the reduction in the total volume of work performed by the G120 group. Despite the lower total workload of training of the G120 group, the animals had similar aerobic capacity responses as other trained groups. Additionally, we observed a decrease in training volume during weeks 6 and 11, which was expected as a result of performing the LMT.

### Body mass, food and water intake

The natural growth of animals can be illustrated by the body mass values throughout the progression of experimental procedures (Fig 4A). From the pre-training period to the end of the taper period, all animals gained ~84% of their body mass. However, animals of the GC group had significant higher levels of body mass starting on the 6^th^ week of training, which persisted until the end of the experimental period. Thus, all different training models prevented the exacerbated gains in body mass that was observed in sedentary animals (GC). Exercise prevents obesity by enhancing the resting metabolism of animals and reducing food intake. Nevertheless, neither food nor water intake were significantly affected by exposure to training. Thus, we believe that the energy expenditure associated with physical training and alterations in the resting metabolisms of animals were the main factors that prevented increase in body mass.

In accordance with body mass results, subjecting animals to all training models positively influenced adipose tissue profiles. Trained animals had significantly reduced levels of retroperitoneal adipose tissue and increased BAT levels in comparison to animals of the GC group. These results are of great interest to health and/or performance-related experimental researchers. Since we were able to demonstrate that regardless of intensity or workload variability throughout training, chronic exercise reduces weight gain and levels of adipose tissue, which indicates that the body compositions of trained animals have improved ratios of lean and fat tissue. It has also been showed that BAT levels play a significant role in thermogenesis, metabolic regulation and body mass control. As a result, researchers have assessed whether altering diet [24] or taking drugs [25] may be efficient methods for increasing levels of the tissue. Enhancing adipose tissue profiles reduces the risk of obesity, diabetes and cardiovascular diseases [26]. Here, we have demonstrated that both non-periodized and periodized training significantly enhance white and brown adipose tissue profiles.

### Blood biomarkers and glycogen stores

Increased albumin levels were found in both G120 and GPE groups. Albumin represents 50% of the total protein in the bloodstream, and plays an important role in the control of osmotic pressure, hormone levels and free fat acid (FFA) transport [27]. Therefore albumin significantly regulates metabolism during exercise, especially with regard to the oxidation of fats. Indeed, we observed that levels of LDL and FFA were significantly reduced in the GPE group, suggesting that periodized training was most effective for enhancing lipid metabolism. This reduced the availability of lipids not only in adipose tissue, but also in the blood stream. These results has implications that affect both health and performance, since lipid levels are directly related to cardiovascular disease risk and the occurrence of metabolic syndromes [21].

Despite the fact that altered levels of LDH were not observed, CK levels of all trained groups were significantly less than those of the GC group. In both humans and animals, blood CK activity can be most prominent 24–72h after muscle damage [28, 29]. Thus, elevated CK activity in the GC group may have occurred as a result of the LMT that occurred 24 h before euthanasia. Interestingly, animals of the GC group could not complete the LMT, and reached exhaustion after 2 or 3 stages of incremental phase. Trained animals completed an average of 5 stages. Therefore, trained animals performed a much greater volume of exercise (LMT) than the GC group, and were determined to possess lower levels of muscle damage markers. This indicated that the training models studied had protective effects.

Uric acid (UA) levels were also reduced in all trained animals when compared to animals of the GC group. UA is a metabolite of purine, which is a nucleotide that comprises DNA, coenzymes like NAD^+^ and the adenosine molecule in ATP. During exhaustive exercises, the inadequate ADP phosphorylation leads to increased levels of inositol monophosphate, which is then further converted to UA [30]. Therefore, reduced levels of UA in trained animals suggests that they have an improved capacity to resynthesize ATP, and thus, improve their energy supply during exercise.

We investigated the effect of exercise intensity and periodization on glycogen repletion. Hepatic glycogen stores of exclusively the GPE group were significantly increased after training in comparison to levels observed in other groups. Similar results were determined in another study that assessed swimming periodization in rats [2]. Increased levels of glycogen stores have also been reported after non-periodized training at intensities near AnT [4]. In the present study, glycogen stores of groups subjected to non-periodized training were increased, however, the differences were not significantly greater than those of the control group. In the other hand, hepatic glycogen levels of the GPE were increased. This confirms the hypothesis that a variable workload during training can optimize the physiological adaptation to chronic exercise, and therefore, should be considered in experimental studies.

### MCT gene and protein expression levels

Monocarboxylate transporters 1 and 4 were chosen as variables of interest due to their close relationship with pH regulation during exercise and metabolic adaptation in response to training [9], which can directly influence aerobic capacity [31]. Despite the fact that we acknowledge the importance of characterizing expression levels of exercise-induced MCT genes, studying the genes is difficult because pre- and post- transcriptional and translational levels MCTs can be affected by multiple factors including hormone release, metabolic disturbance, energy status and even hypoxia [11, 32]. Thus, the present study focused on assessing the effect of exercise volume and intensity on MCT gene and protein expression levels, which occurred regardless of the molecular mechanism used to produce the changes observed.

Several studies have reported that gene expression and protein levels of MCTs increased as a result of exercise [12, 33]. Training models assessed previously included endurance [34], strength [35, 36] and interval training [37]. Some authors observed that MCT1 was most responsive to exercise training at varied intensities [32, 34], while MCT4 expression is most responsive to higher intensity efforts [37]. It is possible that the different response of the two MCT genes to exercise stimulus is associated with their differing roles in the regulation of metabolism. For instance, MCT1 may regulate aerobic metabolism as a result of its high affinity for lactate capture, which provides a substrate for oxidation in muscle and gluconeogenesis in the liver [12, 38]. In the other hand, MCT4 is more abundant in sarcolemma of glycolytic fibers and plays an important role in the regulation of pH throughout high-intensity exercise [33]. However, the nature of the relationship between training intensity MCTs expression remain unclear.

MCT mRNA levels were altered in both trained and control animals, which may suggest that expression of the gene is age-dependent. MCT1 mRNA increased in a tissue- and intensity-dependent manner due to increased levels were observed in the gastrocnemius of animals of the G80 group. However, protein content in gastrocnemius was not altered. In contrast, MCT1 protein content was significantly increased in animals not subjected to periodized training in soleus muscle, even when MCT1 mRNA levels remained unaltered in the tissue. These results are in accordance with literature reports that have shown that regulation of MCTs may occur post-transcriptionally, and thus, protein content is not completely dependent in increased levels of gene expression [32, 39]. Additionally, despite the fact that trained animals were subjected to the same total workload, GPE had the most significantly reduced levels of MCT1 proteins when compared to non-periodized groups. In an interesting review, Thomas et al. [11] reported that the later the biopsy timing, the higher values for the MCT1 protein content, which could trigger the effect of supercompensation. However, this effect should be tested after tapering period.

Therefore, we tested whether reduced workloads that occur throughout the tapering period reduced MCT1 content and observed that levels post-taper were decreased. This suggests that the regulation of MCT1 content is transitory and dependent on the last training session experienced by the animal. Nevertheless, the reduction did not affect the aerobic performance (AnT) or physiological parameters of the GPE group.

Finally, our results corroborate the literature regarding three conjectures: i) the expression of the MCT1 gene and protein are more sensitive to training than MCT4; ii) the protein content did not follow the corresponding gene expression, although our experimental design does not included a time course response; and iii) different intensities and training models affect the expression of the MCT1 gene and its protein content.

## Conclusion

According to our results, all training models successfully prevented typical decreases in aerobic capacity that are commonly observed in animals with sedentary lifestyles or age. At the molecular level, MCT1 isoform was more sensitive to physical training than MCT4, with mRNA increasing in G80 and protein content in all non-periodized trained groups. Further, all training models prevented body mass and fat gain, and reduced the appearance of muscle damage markers and purine metabolites. Apparently, training at different intensities but at same total workload promotes similar adaptations. Nevertheless, despite similar total workload, animals of the GPE group had significantly reduced FFA and LDL levels, and increased hepatic glycogen stores. Although observed differences were modest, these results suggest that periodization models may be used to optimize the physiological responses of rats to swimming training.

## Supporting information

S1 File (PDF)

## References

[1] Coffey VG, Hawley JA. The molecular bases of training adaptation. Sport Med [Internet]. 2007/8/29 2007;37:737–63. Available from: http://www.ncbi.nlm.nih.gov/pubmed/17722947 10.2165/00007256-200737090-00001 17722947

[2] de Araujo GG, Papoti M, Dos Reis IG, de Mello MA, Gobatto CA. Physiological responses during linear periodized training in rats. Eur J Appl Physiol [Internet]. 2011/6/18 2012;112:839–52. Available from: http://www.ncbi.nlm.nih.gov/pubmed/21681481 10.1007/s00421-011-2020-2 21681481

[3] Booth FW, Laye MJ, Spangenburg EE. Gold standards for scientists who are conducting animal-based exercise studies. J Appl Physiol [Internet]. 2009/7/04 2010;108:219–21. Available from: http://www.ncbi.nlm.nih.gov/pubmed/19574508 10.1152/japplphysiol.00125.2009 19574508

[4] de Araujo GG, Papoti M, Delbin MA, Zanesco A, Gobatto CA. Physiological adaptations during endurance training below anaerobic threshold in rats. Eur J Appl Physiol [Internet]. 2013/3/05 2013; Available from: http://www.ncbi.nlm.nih.gov/pubmed/23456272 10.1007/s00421-013-2616-9 23456272

[5] Mille-Hamard L, Breuneval C, Rousseau AS, Grimaldi P, Billat VL. Transcriptional modulation of mitochondria biogenesis pathway at and above critical speed in mice. Mol Cell Biochem. Springer US; 2015;405:223–32. 25912548 10.1007/s11010-015-2413-3

[6] Gerber T, Borg ML, Hayes A, Stathis CG. High-intensity intermittent cycling increases purine loss compared with workload-matched continuous moderate intensity cycling. Eur J Appl Physiol. 2014;114:1513–20. 10.1007/s00421-014-2878-x 24748529 PMC4048667

[7] MacInnis MJ, Gibala MJ. Physiological adaptations to interval training and the role of exercise intensity. J Physiol [Internet]. 2017;595:2915–30. Available from: http://doi.wiley.com/10.1113/JP273196 10.1113/JP273196 PMC5407969 27748956

[8] Rhea MR, Alderman BL. A meta-analysis of periodized versus nonperiodized strength and power training programs. Res Q Exerc Sport [Internet]. 2004 [cited 2015 Mar 31];75:413–22. Available from: http://www.mendeley.com/catalog/metaanalysis-periodized-versus-nonperiodized-strength-power-training-programs/ 15673040 10.1080/02701367.2004.10609174

[9] de Araújo GG, Papoti M, Manchado-Gobatto FB, Mello MAR, Gobatto CA. Padronização de um Protocolo Experimental de Treinamento Periodizado em Natação Utilizando Ratos Wistar. Rev Bras Med do Esporte. 2010;16:51–6.

[10] Felmlee MA, Jones RS, Rodriguez-Cruz V, Follman KE, Morris ME. Monocarboxylate transporters (SLC16): Function, regulation, and role in health and disease. Pharmacol Rev. 2020;72:466–85. 10.1124/pr.119.018762 32144120 PMC7062045

[11] Thomas C, Bishop DJ, Lambert K, Mercier J, Brooks G a. Effects of acute and chronic exercise on sarcolemmal MCT1 and MCT4 contents in human skeletal muscles: current status. AJP Regul Integr Comp Physiol. 2012;302:R1–14. 22012699 10.1152/ajpregu.00250.2011

[12] Brooks GA. The Science and Translation of Lactate Shuttle Theory. Cell Metab [Internet]. Elsevier Inc.; 2018;27:757–85. Available from: 10.1016/j.cmet.2018.03.008 29617642

[13] Beck WR, Botezelli JD, Pauli JR, Ropelle ER, Gobatto CA. Melatonin Has An Ergogenic Effect But Does Not Prevent Inflammation and Damage In Exhaustive Exercise. Sci Rep [Internet]. Nature Publishing Group; 2015;5:18065 Available from: http://www.scopus.com/inward/record.url?eid=2-s2.0-84950298487&partnerID=tZOtx3y1 10.1038/srep18065 PMC4680866 26669455

[14] Rodrigues NA, Torsoni AS, Fante T, dos Reis IGM, Gobatto CA, Manchado-Gobatto FB. Lactate minimum underestimates the maximal lactate steady-state in swimming mice. Appl Physiol Nutr Metab. 2017;42:46–52. 10.1139/apnm-2016-0198 28006434

[15] Lima AA, Gobatto CA, Messias LHD, Scariot PPM, Forte LDM, Santin JO, et al Two water environment adaptation models enhance motor behavior and improve the success of the lactate minimum test in swimming rats. Rev Educ Física. 2017;23:1–8. 10.1590/s1980-6574201700si0009

[16] de Araujo GG, Papoti M, Manchado Fde B, de Mello MA, Gobatto CA. Protocols for hyperlactatemia induction in the lactate minimum test adapted to swimming rats. Comp Biochem Physiol A Mol Integr Physiol [Internet]. 2007/10/30 2007;148:888–92. Available from: http://www.ncbi.nlm.nih.gov/pubmed/17964836 10.1016/j.cbpa.2007.09.002 17964836

[17] Messias LHD, Gobatto CA, Beck WR, Manchado-gobatto FB. The Lactate Minimum Test: Concept, Methodological Aspects and Insights for Future Investigations in Human and Animal Models. Front Physiol. 2017;8:1–17. 28642717 10.3389/fphys.2017.00389 PMC5463055

[18] Regouw BJM, Cornelissen PJHC, Helder RAP, Spijkers JBF, Weeber YMM. Specific determination of free fatty acid in plasma. Clin Chim Acta. 1971;31:187–95. 10.1016/0009-8981(71)90377-9 5544048

[19] Engel PC, Jones JB. Causes and elimination of erratic blanks in enzymatic metabolite assays involving the use of NAD+ in alkaline hydrazine buffers: improved conditions for the assay of L-glutamate, L-lactate, and other metabolites. Anal Biochem [Internet]. 1978/8/01 1978;88:475–84. Available from: http://www.ncbi.nlm.nih.gov/pubmed/29519 10.1016/0003-2697(78)90447-5 29519

[20] Dubois M, Gilles K, Hamilton JK, Rebers PA, Smith F. A colorimetric method for the determination of sugars. Nature [Internet]. 1951/7/28 1951;168:167 Available from: http://www.ncbi.nlm.nih.gov/pubmed/14875032 10.1038/168167a0 14875032

[21] Scariot PPM, Manchado-Gobatto F de B, Torsoni AS, Torsoni MA, Dos Reis IGM, Beck WR, et al Wide housing space and chronic exercise enhance physical fitness and adipose tissue morphology in rats. Appl Physiol Nutr Metab [Internet]. 2015 [cited 2015 Jun 24];40:489–92. Available from: http://www.ncbi.nlm.nih.gov/pubmed/25906078 10.1139/apnm-2014-0416 25906078

[22] Scariot PPM, Manchado-Gobatto F de B, Torsoni AS, dos Reis IGM, Beck WR, Gobatto CA. Continuous Aerobic Training in Individualized Intensity Avoids Spontaneous Physical Activity Decline and Improves MCT1 Expression in Oxidative Muscle of Swimming Rats. Front Physiol [Internet]. 2016;7:1–10. Available from: http://journal.frontiersin.org/Article/10.3389/fphys.2016.00132/abstract 27148071 10.3389/fphys.2016.00132 PMC4834519

[23] Svedahl K, MacIntosh BR. Anaerobic threshold: the concept and methods of measurement. Can J Appl Physiol [Internet]. 2003/6/27 2003;28:299–323. Available from: http://www.ncbi.nlm.nih.gov/pubmed/12825337 10.1139/h03-023 12825337

[24] Saito M, Yoneshiro T, Matsushita M. Activation and recruitment of brown adipose tissue by cold exposure and food ingredients in humans. Best Pract Res Clin Endocrinol Metab [Internet]. Elsevier Ltd; 2016;1–11. Available from: http://linkinghub.elsevier.com/retrieve/pii/S1521690X1630046X 10.1016/j.beem.2016.08.003 27697214

[25] Mukherjee J, Baranwal A, Schade KN. Classification of Therapeutic and Experimental Drugs for Brown Adipose Tissue Activation: Potential Treatment Strategies for Diabetes and Obesity. Curr Diabetes Rev [Internet]. 2016 [cited 2016 Sep 19]; Available from: http://www.ncbi.nlm.nih.gov/pubmed/27183844 10.2174/1573399812666160517115450 PMC5425649 27183844

[26] Blondin DP, Carpentier AC. The role of BAT in cardiometabolic disorders and aging. Best Pract Res Clin Endocrinol Metab [Internet]. 2016; Available from: http://linkinghub.elsevier.com/retrieve/pii/S1521690X16300501 27697211 10.1016/j.beem.2016.09.002

[27] Romijn JA, Coyle EF, Sidossis LS, Gastaldelli A, Horowitz JF, Endert E, et al Regulation of endogenous fat and carbohydrate metabolism in relation to exercise intensity and duration. Am J Physiol—Endocrinol Metab. 1993;265:E380–391. 8214047 10.1152/ajpendo.1993.265.3.E380

[28] Bessa AL, Oliveira VN, Agostini GG, Oliveira RJS, Oliveira ACS, White GE, et al Exercise Intensity and Recovery: Biomarkers of Injury, Inflammation, and Oxidative Stress. J Strength Cond Res [Internet]. 2016;30:311–9. Available from: http://www.ncbi.nlm.nih.gov/pubmed/23604000 10.1519/JSC.0b013e31828f1ee9 23604000

[29] Huang K-C, Chiu Y-H, Liao K-W, Ke C-Y, Lee C-J, Chao Y-FC, et al Prophylactic acetylsalicylic acid attenuates the inflammatory response but fails to protect exercise-induced liver damage in exercised rats. Eur J Pharmacol [Internet]. Elsevier; 2016;786:204–11. Available from: http://linkinghub.elsevier.com/retrieve/pii/S0014299916303570 27262381 10.1016/j.ejphar.2016.05.043

[30] Kuipers H. Training and overtraining: an introduction. Med Sci Sports Exerc [Internet]. 1998 [cited 2016 Sep 26];30:1137–9. Available from: http://www.ncbi.nlm.nih.gov/pubmed/9662685 10.1097/00005768-199807000-00018 9662685

[31] Billat VL, Sirvent P, Py G, Koralsztein JP, Mercier J. The concept of maximal lactate steady state: a bridge between biochemistry, physiology and sport science. Sport Med [Internet]. 2003/5/15 2003;33:407–26. Available from: http://www.ncbi.nlm.nih.gov/pubmed/12744715 10.2165/00007256-200333060-00003 12744715

[32] Bonen A, Tonouchi M, Miskovic D, Heddle C, Heikkila JJ, Halestrap AP. Isoform-specific regulation of the lactate transporters MCT1 and MCT4 by contractile activity. Am J Physiol Endocrinol Metab [Internet]. 2000/10/29 2000;279:E1131–8. Available from: http://www.ncbi.nlm.nih.gov/pubmed/11052969 10.1152/ajpendo.2000.279.5.E1131 11052969

[33] Bisetto S, Wright MC, Nowak RA, Lepore AC, Khurana TS, Loro E, et al New Insights into the Lactate Shuttle: Role of MCT4 in the Modulation of the Exercise Capacity. iScience [Internet]. Elsevier Inc.; 2019;22:507–18. Available from: 10.1016/j.isci.2019.11.041 31837519 PMC6920289

[34] Dubouchaud H, Butterfield GE, Wolfel EE, Bergman BC, Brooks G a. Endurance training, expression, and physiology of LDH, MCT1, and MCT4 in human skeletal muscle. Am J Physiol Endocrinol Metab. 2000;278:E571–9. 10.1152/ajpendo.2000.278.4.E571 10751188

[35] Juel C, Holten MK, Dela F. Effects of strength training on muscle lactate release and MCT1 and MCT4 content in healthy and type 2 diabetic humans. J Physiol. 2004;556:297–304. 10.1113/jphysiol.2003.058222 14724187 PMC1664883

[36] Pilegaard H, Domino K, Noland T, Juel C, Hellsten Y, Halestrap AP, et al Effect of high-intensity exercise training on lactate/H+ transport capacity in human skeletal muscle. Am J Physiol Endocrinol Metab. 1999;276:255–61. 9950784 10.1152/ajpendo.1999.276.2.E255

[37] Juel C, Klarskov C, Nielsen JJ, Krustrup P, Mohr M, Bangsbo J. Effect of high-intensity intermittent training on lactate and H+ release from human skeletal muscle. Am J Physiol Endocrinol Metab [Internet]. 2003/10/16 2004;286:E245–51. Available from: http://www.ncbi.nlm.nih.gov/pubmed/14559724 10.1152/ajpendo.00303.2003 14559724

[38] Droździk M, Szeląg‐pieniek S, Grzegółkowska J, Łapczuk‐romańska J, Post M, Domagała P, et al Monocarboxylate transporter 1 (MCT1) in liver pathology. Int J Mol Sci. 2020;21:1–12. 10.3390/ijms21051606 PMC7084425 32111097

[39] Bickham DC, Bentley DJ, Le Rossignol PF, Cameron-Smith D. The effects of short-term sprint training on MCT expression in moderately endurance-trained runners. Eur J Appl Physiol. 2006;96:636–43. 10.1007/s00421-005-0100-x 16408234

