# Supplementary material for: Periodized versus non-periodized swimming training with equal total training load: Physiological, molecular and performance adaptations in Wistar rats
Source: PLoS One. 2020 Sep 30;15(9):e0239876. doi: 10.1371/journal.pone.0239876 (PMC7526899; doi:10.1371/journal.pone.0239876)

Obs: All blots used in figures were horizontally inverted in paper to meet the proper sequence of groups. The red "X" identifies lanes not used to illustrate figures in paper.

SOLEUS

MCT1 (membrane 1)

43 kDa

(used in Figure 9)

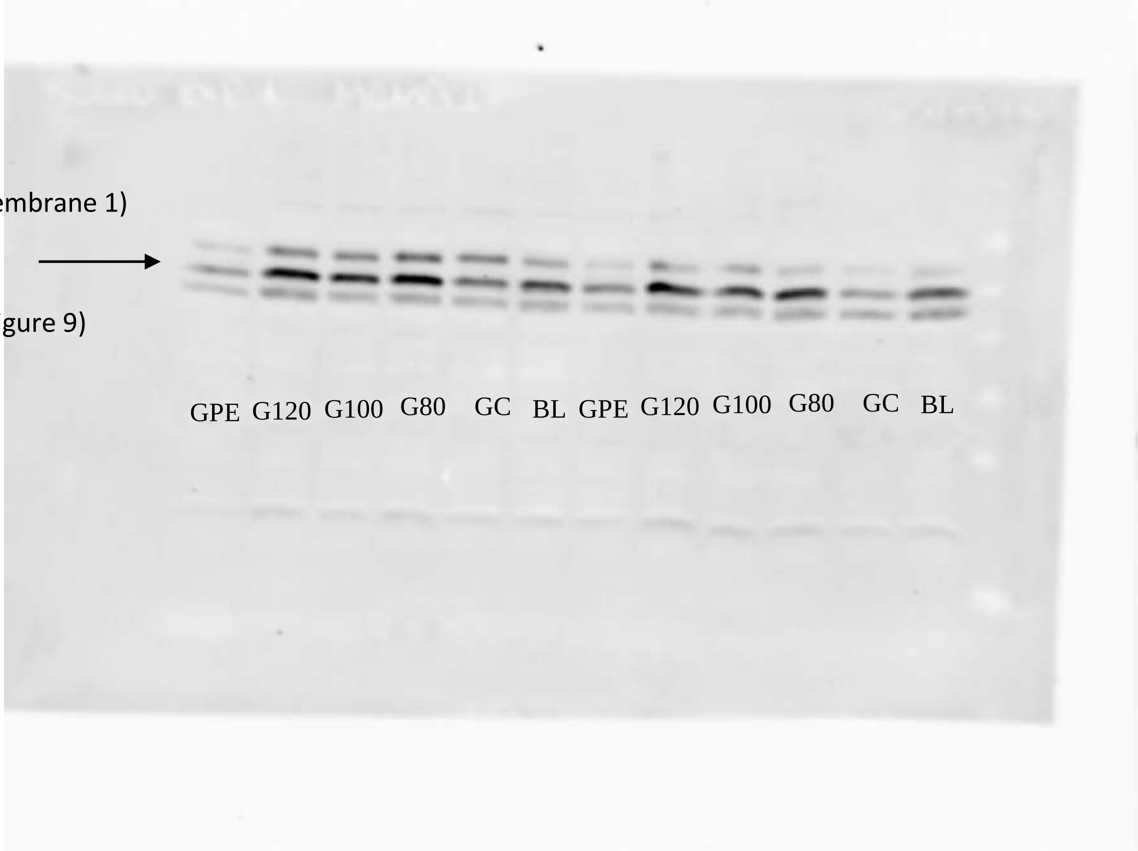

a-tubulin (for MCT1)
52 kDa

(membrane 1)

(used in Figure 9)

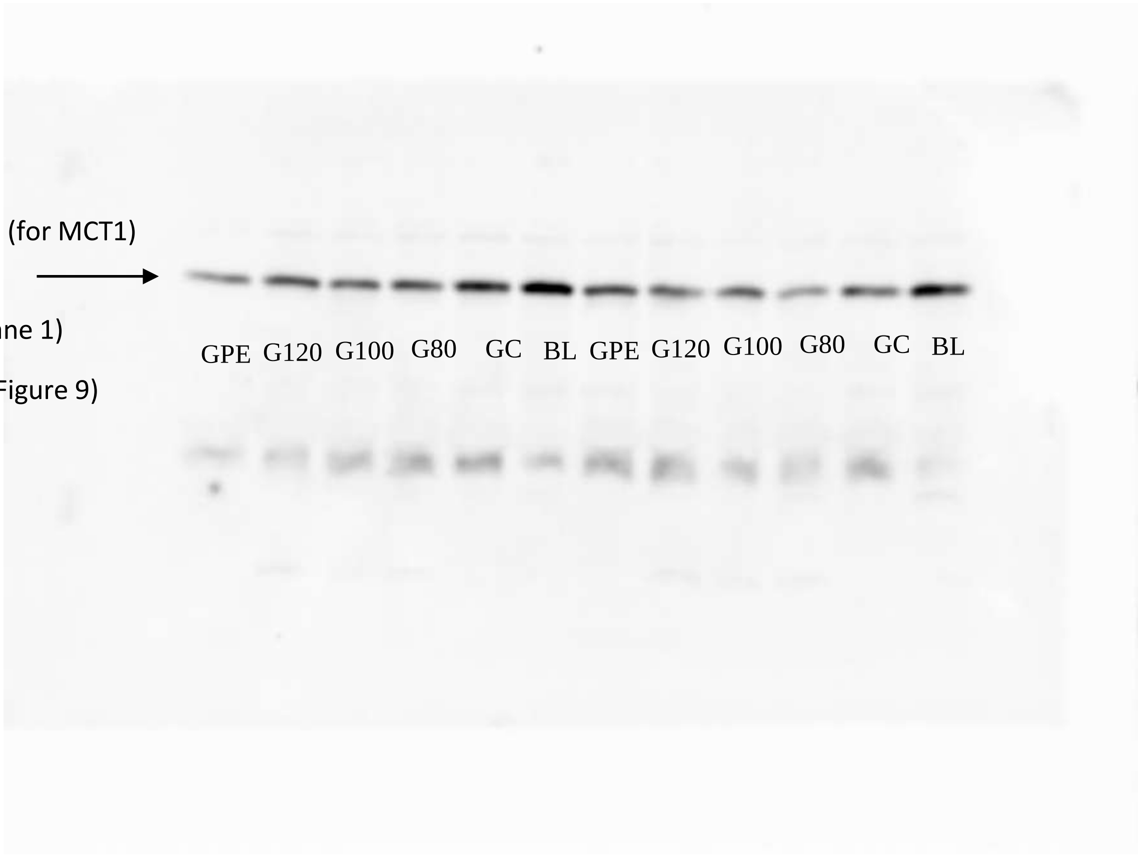

MCT1 (membrane 2)

43 kDa

Not used in figures, but pooled on statistics analysis

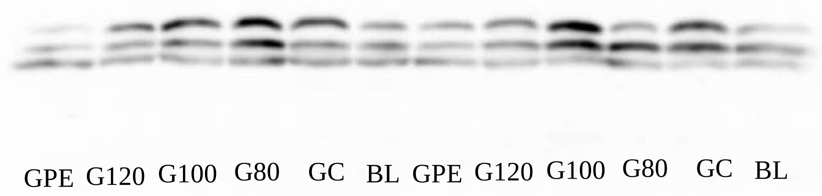

a-tubulin (for MCT1) (membrane 2)

52kDa

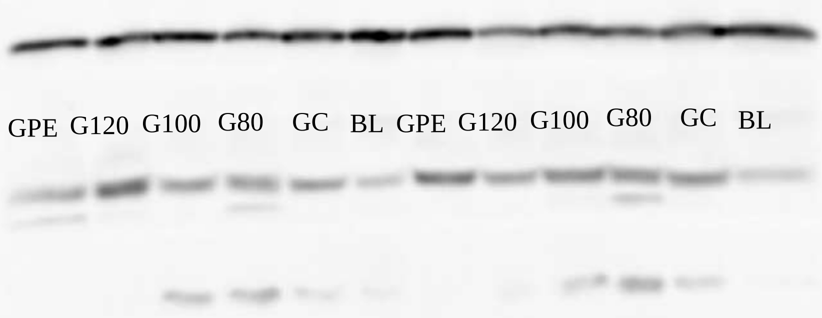

MCT1 (membrane 3)

43kDa

Not used in figures, but pooled on statistics analysis

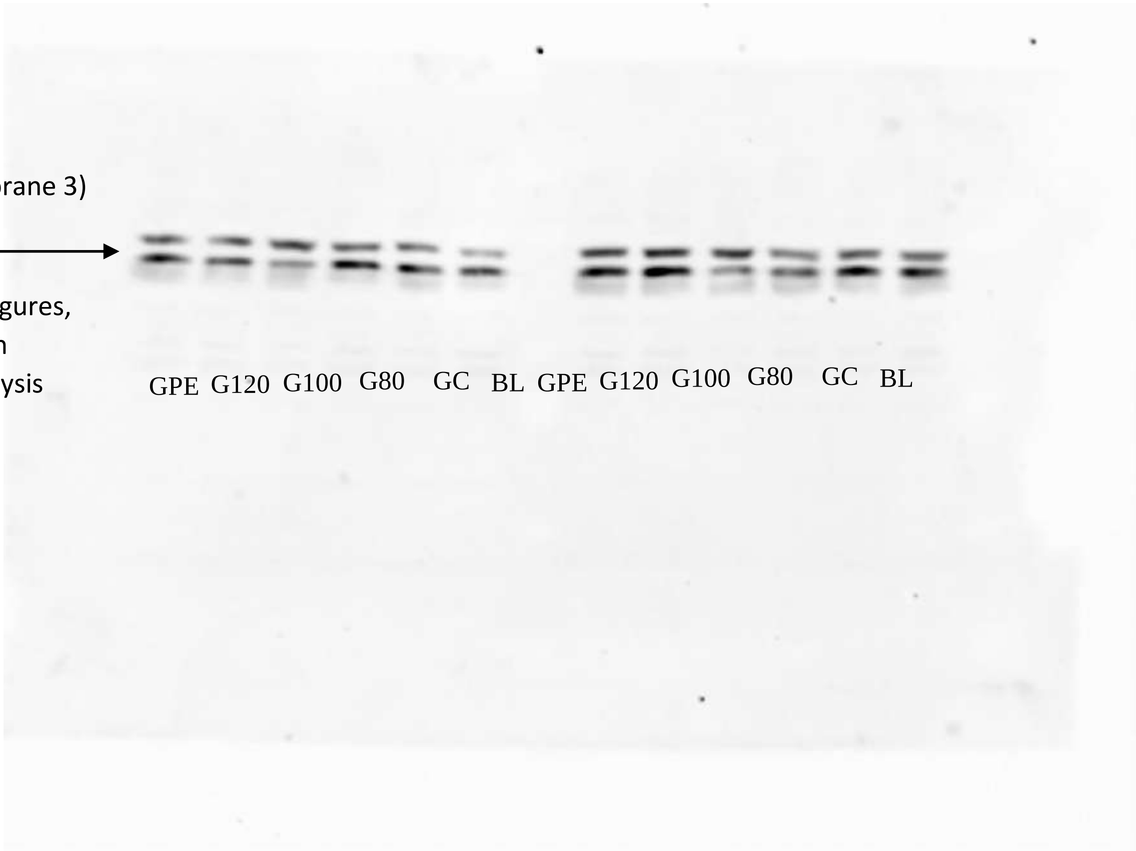

a-tubulin (for MCT1) (membrane 3)

52kDa

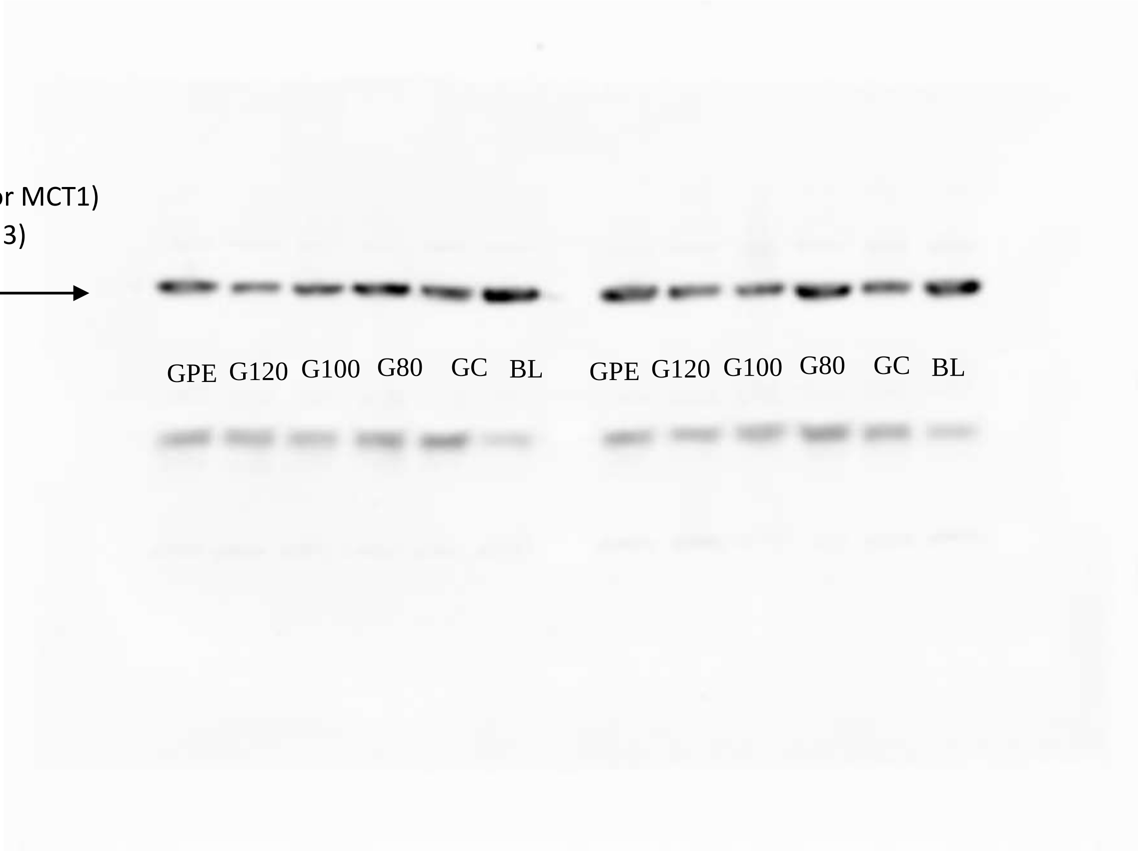

MCT4 (membrane 1)

43kDa

Not used in figures, but pooled on statistics analysis

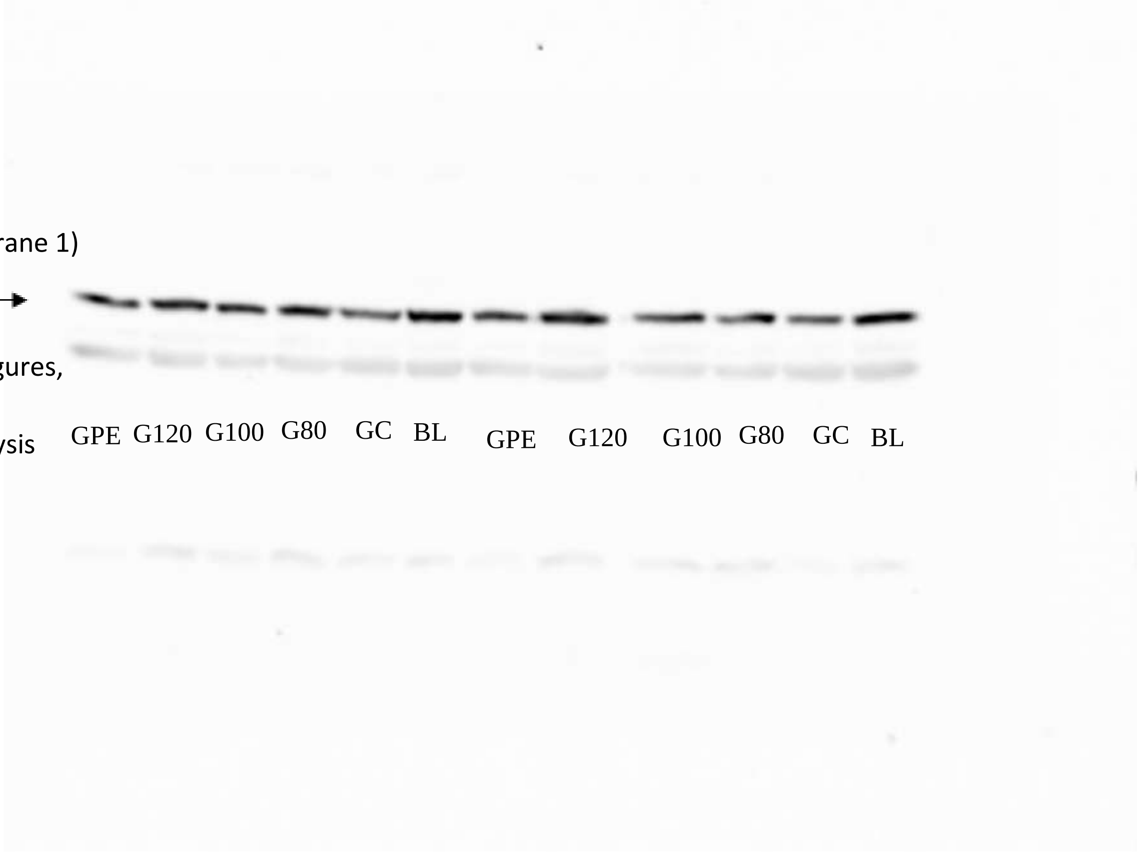

a-tubulin (for MCT4) (membrane 1)

52kDa

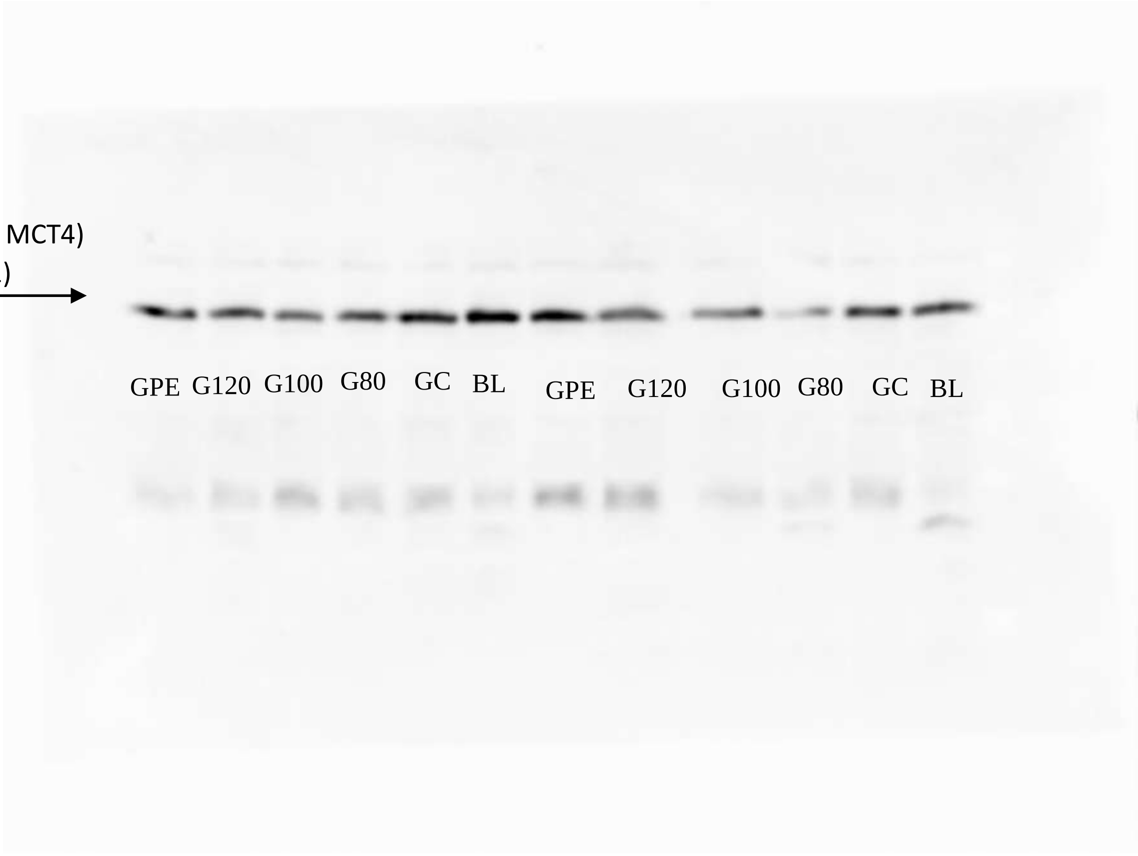

MCT4 (membrane 2)

43kDa

used in Figure 10

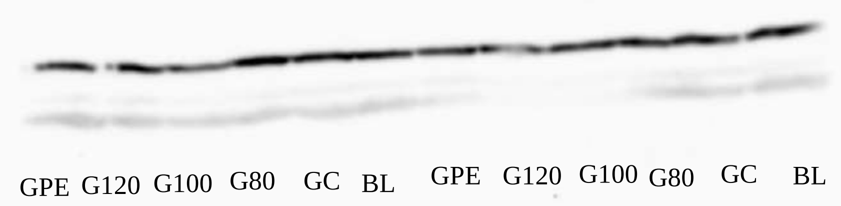

a-tubulin (for MCT4)
(membrane 2)

52kDa

Used in Figure 10

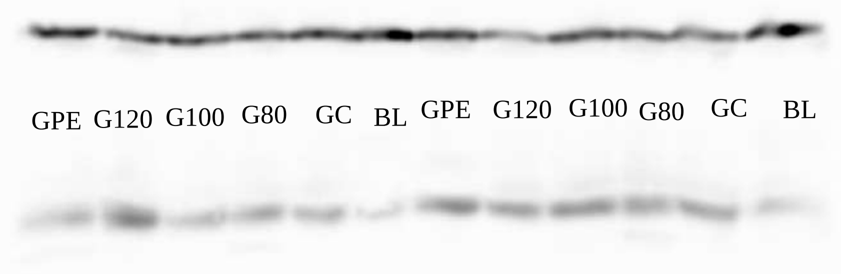

MCT4 (membrane 3)

43kDa

Not used in figures, but pooled on statistics analysis

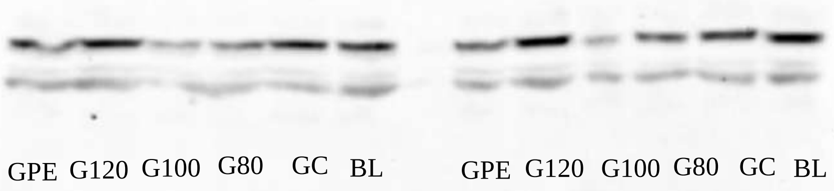

a-tubulin (for MCT4) (membrane 3)

52kDa

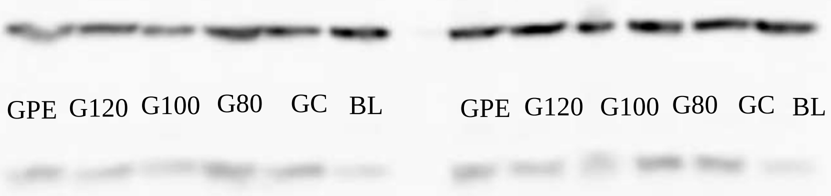

GASTROCNEMIUS

MCT1 (membrane 1)

43kDa

Used in Figure 9

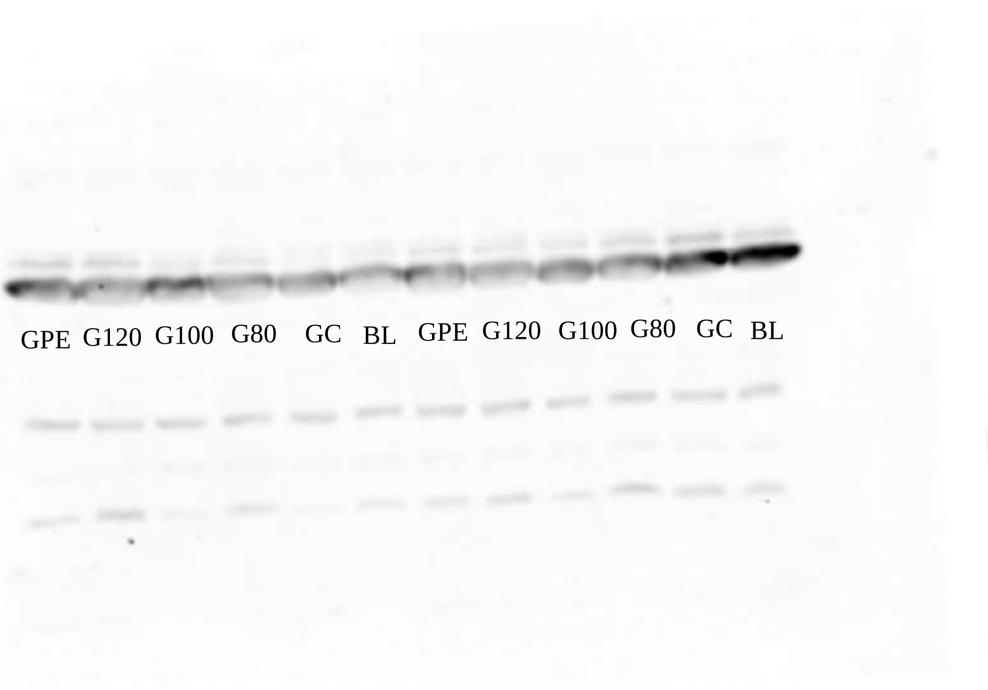

a-tubulin (for MCT1)
(membrane 1)

52kDa

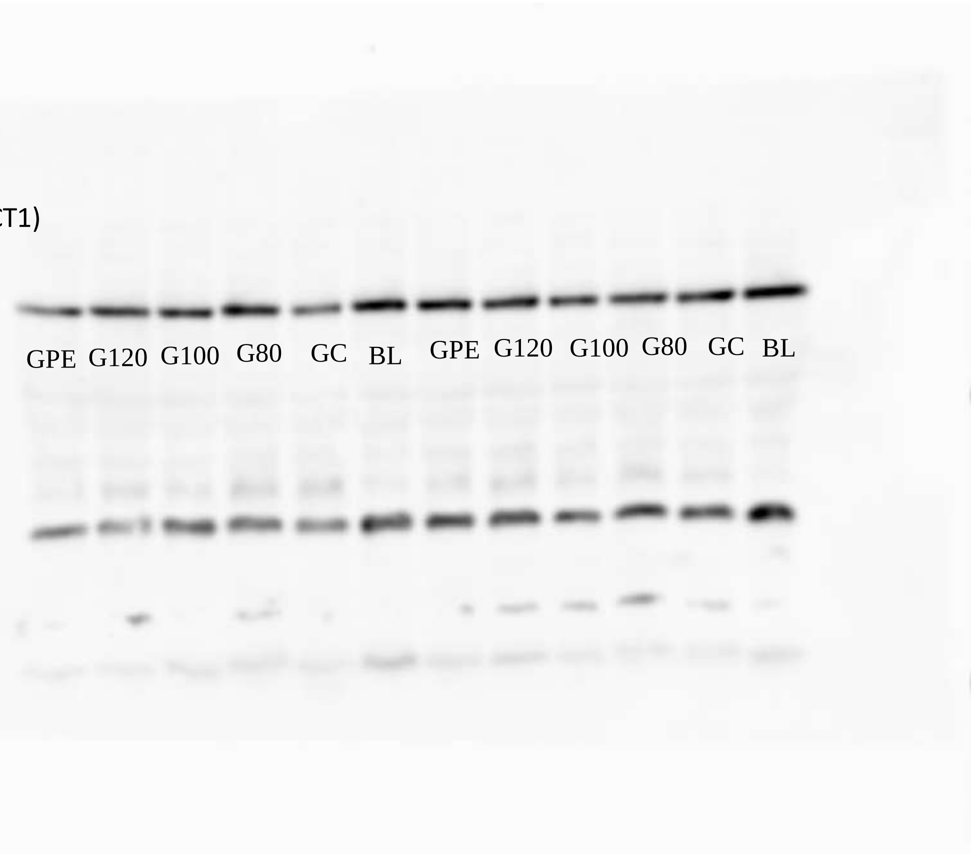

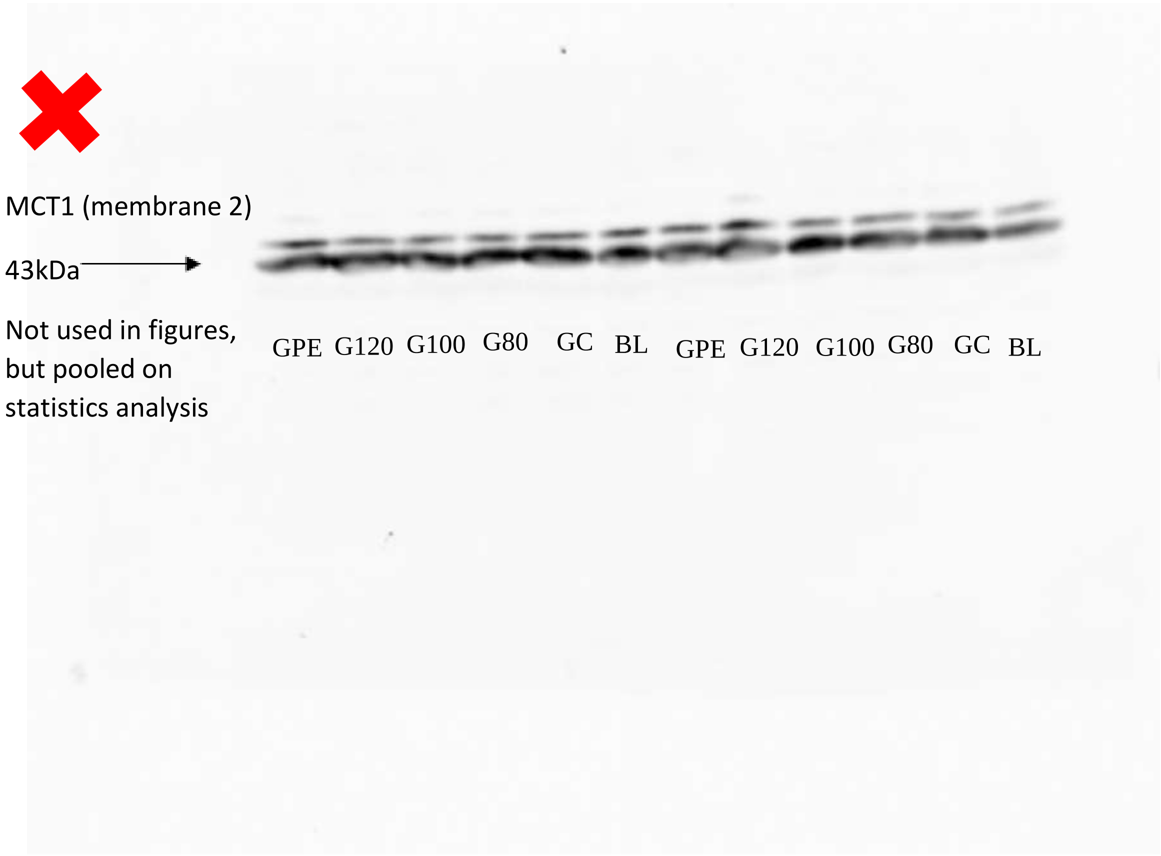
MCT1 (membrane 2)
43kDa
Not used in figures, but pooled on statistics analysis
GPE G120 G100 G80 GC BL GPE G120 G100 G80 GC BL

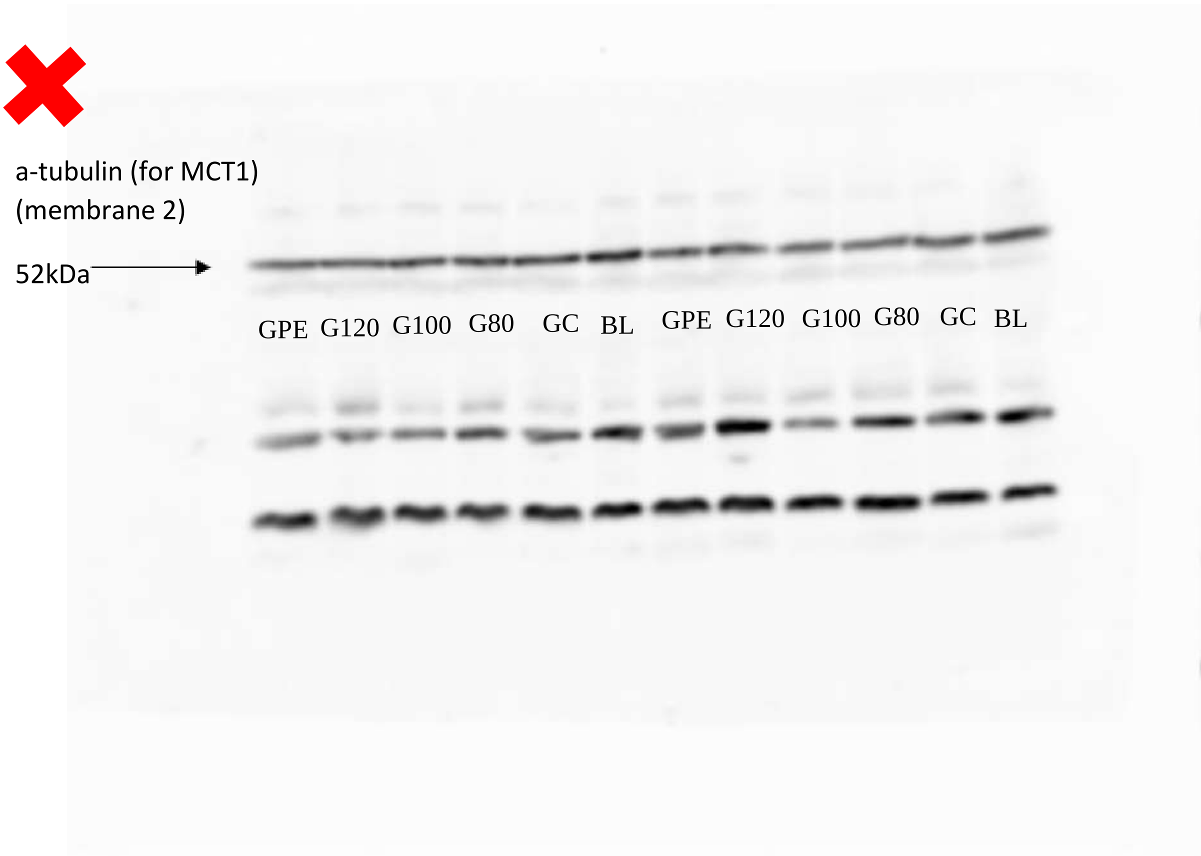
a-tubulin (for MCT1) (membrane 2)
52kDa
GPE G120 G100 G80 GC BL GPE G120 G100 G80 GC BL

MCT1 (membrane 3)

43kDa

Not used in figures, but pooled on statistics analysis

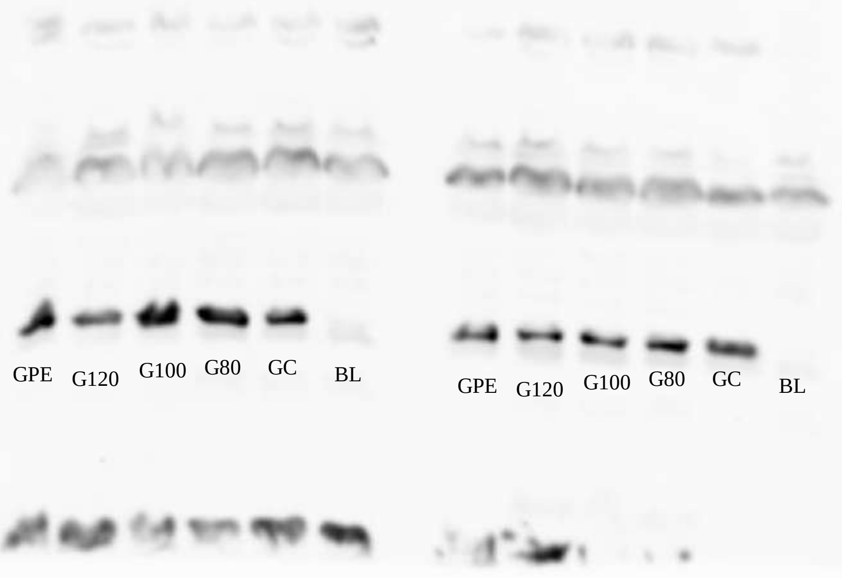

a-tubulin (for MCT1) (membrane 3)

52kDa

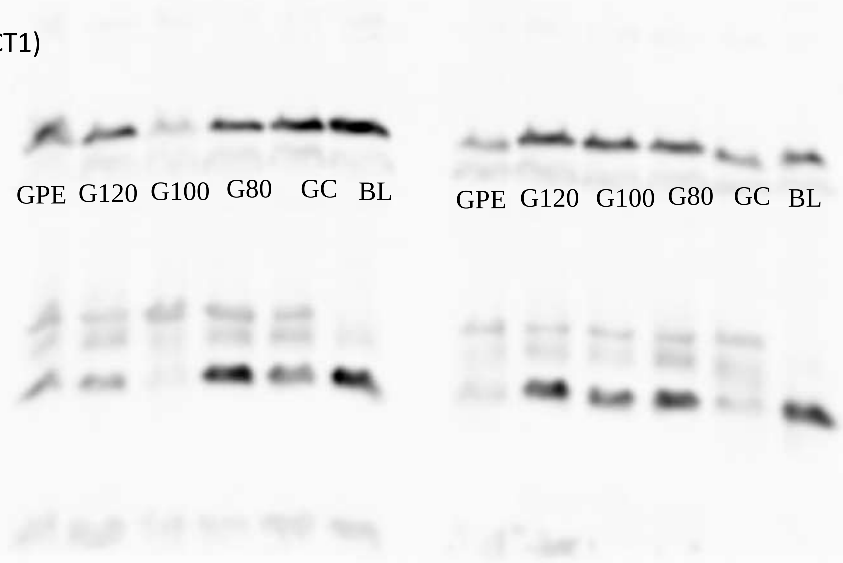

MCT4 (membrane 1)

43kDa

Not used in figures, but pooled on statistics analysis

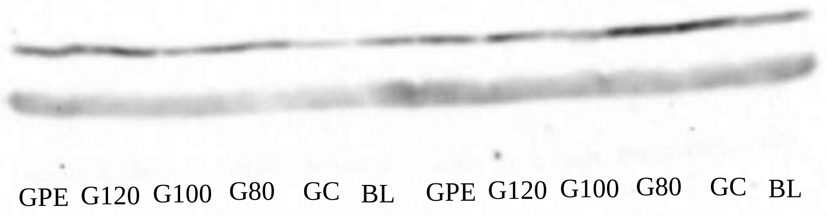

a-tubulin (for MCT4) (membrane 1)

52kDa

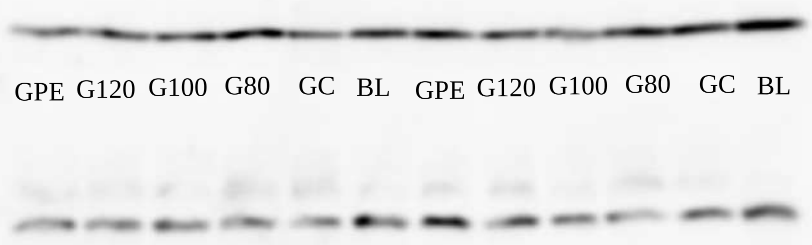

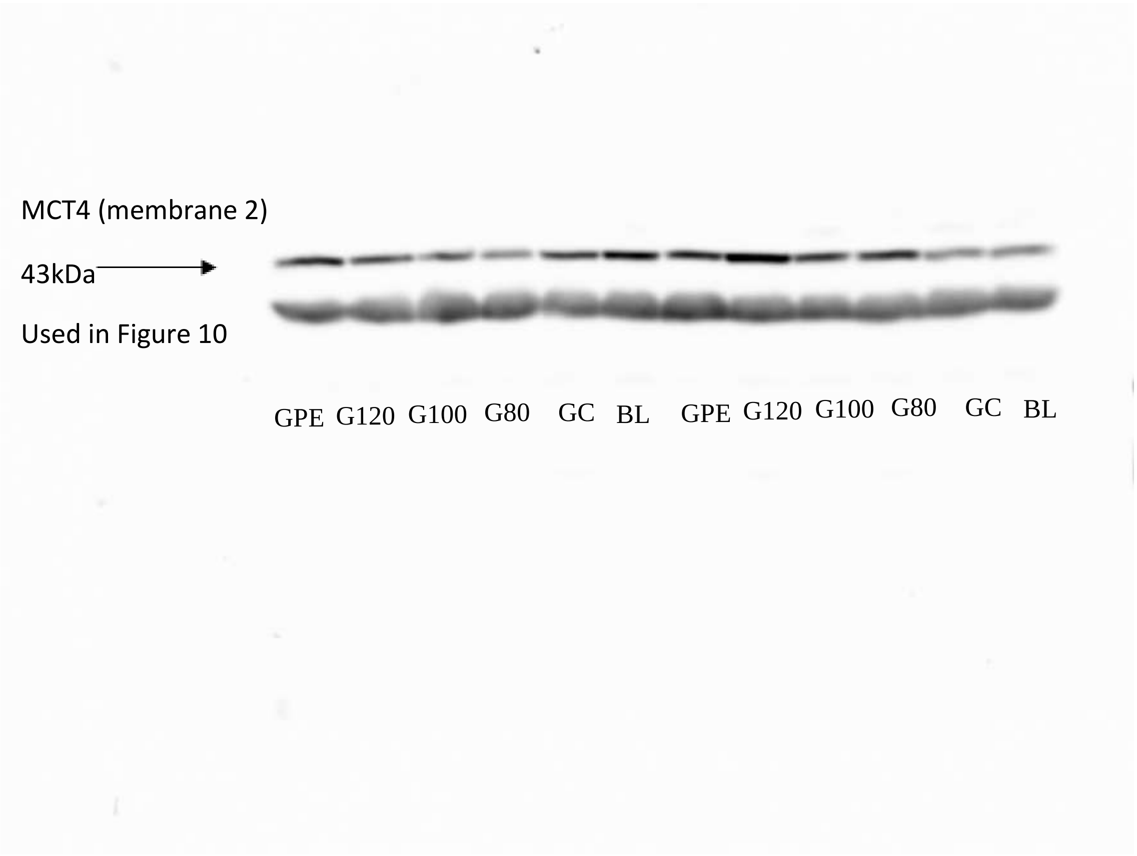
MCT4 (membrane 2)
43kDa
Used in Figure 10
GPE G120 G100 G80 GC BL GPE G120 G100 G80 GC BL

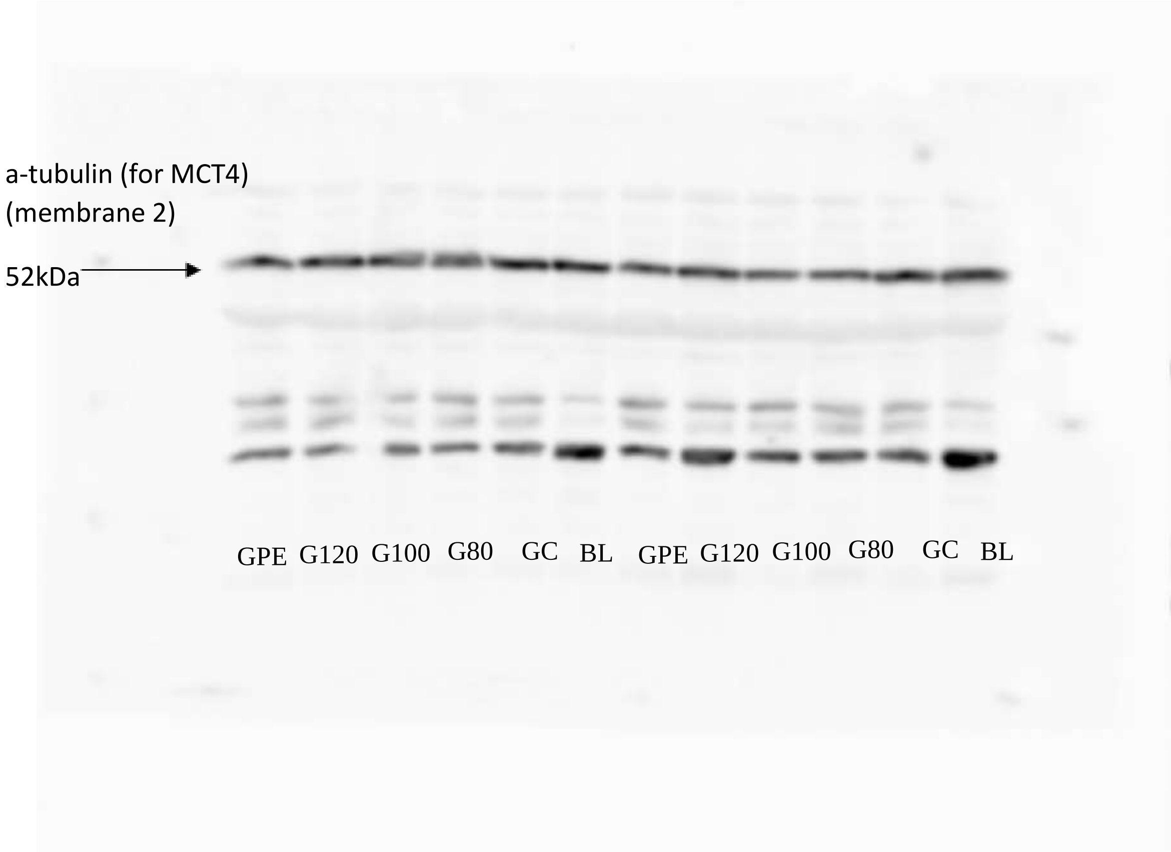
a-tubulin (for MCT4)
(membrane 2)
52kDa
GPE G120 G100 G80 GC BL GPE G120 G100 G80 GC BL

MCT4 (membrane 3)

43kDa

Not used in figures

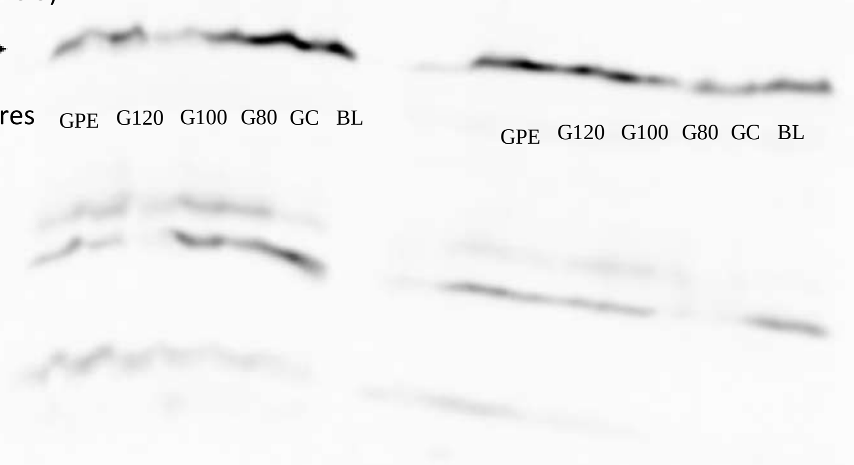

a-tubulin (for MCT4)
(membrane 3)

52kDa

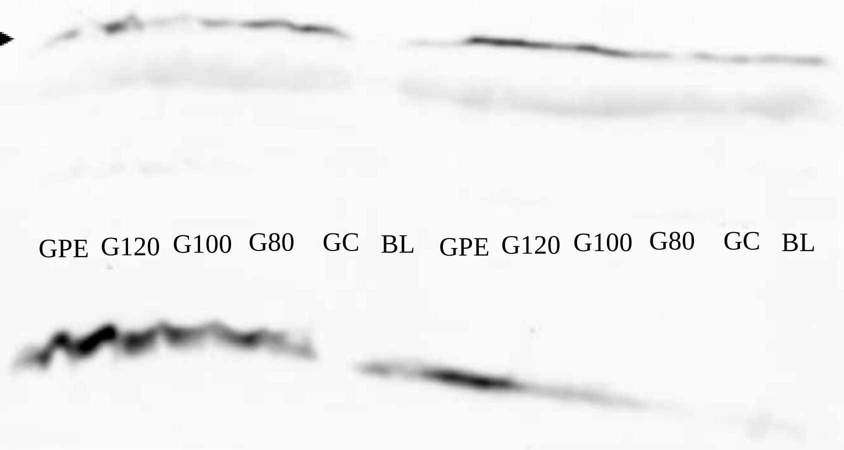

Supplement: S1 File — (PDF) [file pone.0239876.s001.pdf]
